# Chemical Multiverse and Diversity of Food Chemicals

**DOI:** 10.1021/acs.jcim.3c01617

**Published:** 2024-02-15

**Authors:** Juan F. Avellaneda-Tamayo, Ana L. Chávez-Hernández, Diana L. Prado-Romero, José L. Medina-Franco

**Affiliations:** DIFACQUIM Research Group, Department of Pharmacy, School of Chemistry, Universidad Nacional Autónoma de México, Avenida Universidad 3000, Mexico City 04510, Mexico

## Abstract

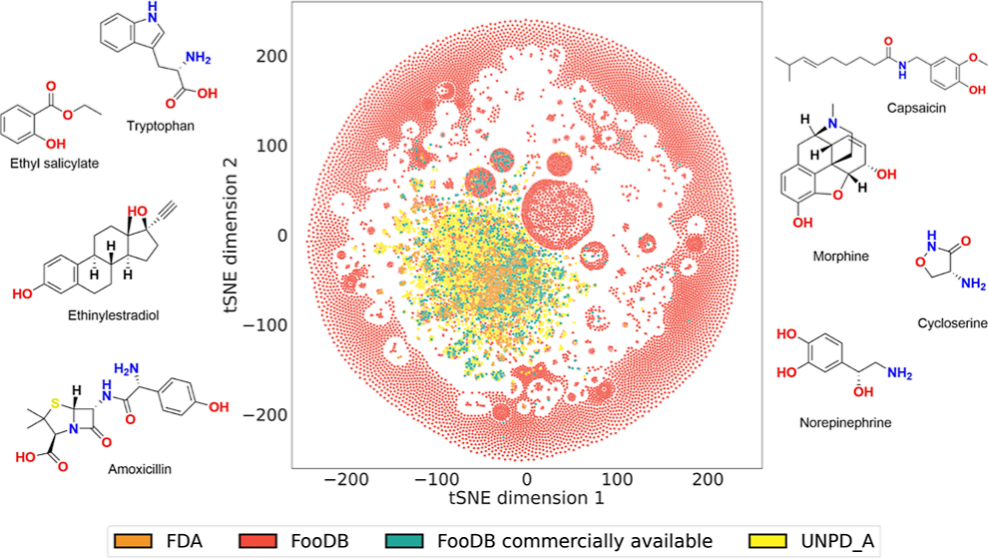

Food chemicals have a fundamental role in our lives, with an extended impact on nutrition, disease prevention, and marked economic implications in the food industry. The number of food chemical compounds in public databases has substantially increased in the past few years, which can be characterized using chemoinformatics approaches. We and other groups explored public food chemical libraries containing up to 26,500 compounds. This study aimed to analyze the chemical contents, diversity, and coverage in the chemical space of food chemicals and additives and, from here on, food components. The approach to food components addressed in this study is a public database with more than 70,000 compounds, including those predicted via *omics* techniques. It was concluded that food components have distinctive physicochemical properties and constitutional descriptors despite sharing many chemical structures with natural products. Food components, on average, have large molecular weights and several apolar structures with saturated hydrocarbons. Compared to reference databases, food component structures have low scaffold and fingerprint-based diversity and high structural complexity, as measured by the fraction of sp^3^ carbons. These structural features are associated with a large fraction of macronutrients as lipids. Lipids in food components were decompiled by an analysis of the maximum common substructures. The chemical multiverse representation of food chemicals showed a larger coverage of chemical space than natural products and FDA-approved drugs by using different sets of representations.

## 1 Introduction

Food chemicals are a rich source of bioactive compounds, including macro- and micronutrients. The human diet is principally based on macronutrients represented by carbohydrates, proteins, and fats. Micronutrients also play a significant role in various physiological and biochemical processes. They are present in fewer amounts in foods, partly due to their energy-expensive plant production.^1^ In addition, many secondary metabolites are mainly responsible for characteristic flavors, colors, and aromas of vegetables, fruits, herbs, and spices^2^ or even processed foods and derivatives as fermented beverages. For example, mezcal, a Mexican traditional beverage, has a distinct composition of compounds such as limonene and pentyl butanoate,^3^ and their identification helps to assess the mezcal’s quality and authenticity.^4^ Other metabolites have been identified as being bioactive and beneficial for human health. For example, flavonoids, carotenoids, and phenolic compounds can act as antioxidants and have been associated with reduced risk of some chronic diseases, including cardiovascular or neurodegenerative disorders.^5−7^ As will be discussed later in this study, many food chemicals are used in the clinic to improve human health.

An increasing number of reports on the structures and bioactivity of isolated compounds from food sources has rapidly raised the registered content in different kinds of food databases, including their applicability. This has also impacted information about the relationship between diet and health. One of these databases is FoodData Central, a public database provided by the U.S. Department of Agriculture.^8^ Besides the chemical composition and nutritional values of foods, it includes information about experimental foods, which are those produced under alternative management systems, experimental genotypes, or research/analytical protocols. FoodData Central comprises information from five distinct data sets.^8^ Another example is FooDB, the largest and most comprehensive public database of food constituents, including food chemicals and additives. Herein, we will refer to the conjunction of these categories as food components.^9^ Content of FooDB is related to compositional, biochemical, physicochemical, and physiological information, including presumptive health effects reported in the literature for both macronutrients and micronutrients, such as secondary metabolites.^9^ As of the current writing, FooDB includes information on 70,926 compounds. 3751 compounds are labeled “detected and quantified”, 11,999 are labeled “detected but not quantified”, 37,384 are “expected but not quantified”, and 17,792 are “predicted”. It is remarkable to clarify that FooDB includes information on metabolomic and lipidomic high-throughput elucidation results, with highly probable structural identification, which gives place to the possible presence of duplicate compounds or inexact elucidation for some others. However, those are well-established and validated experimental and data treatment methods that yield reliable results.^10^

Chemoinformatics analysis on food component databases has been increasingly applied in the past few years. In 2018, Naveja et al. reported a chemoinformatics characterization of FooDB (with 23,883 compounds at the time of that work), analyzing its chemical space coverage and chemical diversity. In that study, FooDB was compared to Generally Recognized as Safe (GRAS) flavoring substances, approved drugs for clinical use, and a random subset of drug-like natural products from ZINC.^11^ That analysis showed that the chemical space of food components, just like their physicochemical properties, overlaps with drugs approved by the U.S. Food and Drug Administration (FDA) and natural products from ZINC. Moreover, FooDB showed a larger structural diversity, as demonstrated by molecular fingerprints, and a higher compound complexity, although a low scaffold diversity.^11^ In a separate study, Kaya and Colmenarejo quantified nuisance substructures in FooDB (26,457 valid compounds at the time of that study), applying pan-assay interference compounds (PAINS),^12^ Invalid Metabolic Panaceas (IMP), and other filters. Those were used as an approximation for promiscuity, false positive and aggregator features, nuisance substructural detection, and a comparative analysis against a set of drugs approved for clinical use. The authors identified 19 different substructures with PAINS alerts; the most frequent matching filters out of 481 were “catechol_A(92)”, “quinone_A(370)”, and “imine_one_A(321)”. In contrast, the subset of approved drugs for clinical use showed 1.1% of compounds with the “catechol_A(92)” moiety, for compounds derived or isolated from natural products. Except for two, all IMP alerts were present in FooDB. While 10 of them were found in DrugBank-approved drugs, the majority of them were in compounds with lower therapeutic value according to the literature. Those facts validate the usage of such applications and also demonstrate their limitations.^13^ These analyses were updated by the same authors with a more recent version of FooDB containing 70,855 compounds. In that work, they found a considerable increment of aliphatic substructures, with the application of Glaxo and LINT alerts.^14^

Aided by natural language processing technology, Zhang et al. curated a comprehensive public database with 12,018 food risk components and characterized them according to their physicochemical properties, scaffold content, compound diversity, and chemical classification.^15^ Additionally, due to its diversity, food components (FooDB) have been effectively used in the generation of publicly available fragment libraries, useful as building blocks for designing bioactive molecules (with 23,883 compounds at the time of that work).^16^

Recently, Sánchez-Ruiz and Colmenarejo reported a model to postulate probable bioactivities of food components by pairing them with compounds from ChEMBL against 19 target classes with a coincidence of 1.6% of FooDB. Through a Similarity Ensemble Approach, they achieved the target assignment of 64.2% of compounds in FooDB.^17^

The aforementioned studies highlight the interest and utility of the systematic analysis of food component databases. Moreover, the size of food components in the public domain is increasing rapidly. A recent characterization of the chemical space of food components is lacking since the size of the database has increased by more than three times in the past few years.

The main goal of this study was to characterize the chemical contents, diversity, and coverage in the chemical space of food components in the latest version of FooDB. We compared the physicochemical properties associated with oral bioavailability and molecular scaffolds of FooDB regarding reference databases of FDA-approved drugs for clinical use and natural products. Data sets were analyzed in terms of structural complexity using the fraction of sp^3^ carbon atoms (CSP3) and their diversity using four molecular fingerprints: Molecular ACCes System (MACCS) keys (166-bits),^18^ extended connectivity fingerprint (ECFP)^19^ of 1024-bits with diameter 4 (ECFP4) and diameter 6 (ECFP6), and MinHashed atom-pair fingerprint up to a diameter of four bonds (MAP4).^20^ New methods for natural product class assignments were incorporated to investigate the exact composition of foods vs natural products. Also, we generated a chemical multiverse visualization of FooDB through different types of molecular representations.^21^ The most frequent molecular scaffolds and structural fragments present in the food components were identified. We also estimate the number of compounds in FooDB that are commercially available. We anticipate that the results presented in this study can boost the identification and development of biologically active compounds in the chemical space of food components. Most of the remarkable differences we found between food components and natural products are due to the overrepresented content of lipids, especially fatty acids in FooDB, in contrast to terpenoids, which are the major class of natural products. This study also represents a step forward toward the growth of the food chemical informatics—foodinformatics—field to ultimately contribute to human health and nutrition.^22^

## 2 Methods

### 2.1 Data Sets

As an approach to the food components’ multiverse, we employed FooDB. As of the current writing, FooDB contains 70,926 compounds from 797 different food sources.^9^ We used the following as reference databases: the FDA set (update until 04 January 2023)^23,24^ with 2324 unique compounds and Universal Natural Product Database—Subset A (UNPD-A) compounds, which includes the 14,994 most diverse compounds of natural products from the UNPD, optimized using the MaxMin algorithm.^25,26^ In addition, a set of commercially available food components was created by pairing FooDB with the available databases in ZINC20,^27^ as described in the Methods Section 2.10.

### 2.2 Data Set Standardization

Compounds in FooDB, UNPD-A, and FDA-approved drugs encoded as Simplified Molecular Input Line Entry System (SMILES)^28^ were standardized using the open-source chemoinformatics toolkit RDKit, version 2022.9.4^29^ and MolVS.^30^ According to the standardization protocol, the functions Standardizer, LargestFragmentChoser, Uncharger, Reionizer, and TautomerCanonicalizer implemented in MolVS^31^ were used. The MolVS TautomerCanonicalizer selects the most logical tautomer from a chemical standpoint for those food molecules with several tautomeric structures of equivalent stability through a scoring system of all potential tautomers. Compounds with valence errors or composed of chemical elements other than H, B, C, N, O, F, Si, P, S, Cl, Se, Br, and I were removed. Stereochemistry information, when available, was kept for analysis such as molecular complexity, natural product likeness (NPL) score, fingerprint-based structural diversity, chemical profiling, and chemical multiverse. Otherwise, the stereochemistry was removed. Compounds with multiple components were split, and the largest component was retained. The remaining compounds were neutralized by adding or subtracting hydrogen atoms to generate the corresponding canonical tautomer.

### 2.3 Data Set Overlap

Overlapping of FooDB with natural products (UNPD-A) and FDA-approved drugs was carried out according to molecular structure and molecular scaffolds (Bemis-Murcko scaffolds, described in Section 2.5). Compound overlap was determined by assessing their canonical SMILES, disregarding chirality.

### 2.4 Molecular Descriptors

For each molecule, physicochemical properties of pharmaceutical interest, constitutional descriptors, and molecular fingerprints were calculated with Python language (using RDKit toolkit), DataWarrior 5.5.0,^32^ and Molecular Operating Environment (MOE), version 2022.02.^33^

Descriptors computed with the RDKit toolkit were hydrogen bond acceptors (HBAs), hydrogen bond donors (HBDs), partition coefficient octanol/water (logP), topological polar surface area (TPSA), molecular weight (MW), CSP3, number of heavy atoms, number of ring systems, number of heteroatoms, number of rotatable bonds (RB), number of alicyclic ring compounds formed by carbon atoms, number of alicyclic rings that include heteroatoms, number of aromatic rings formed by carbon atoms, number of aromatic rings that include heteroatoms, and the total number of aromatic rings. The number of acid atoms, aromatic atoms, basic atoms, nitrogen, oxygen, and halogen atoms, the fraction of RB, the number of chiral centers, and formal charges were computed using the MOE. The DataWarrior complexity index was calculated with DataWarrior 5.5.0.

Four types of molecular fingerprints with different designs were calculated: Molecular ACCes System (MACCS) keys (166-bits),^18^ ECFP^19^ of 1024-bits with diameter 4 (ECFP4) and diameter 6 (ECFP6), and MinHashed atom-pair fingerprint up to a diameter of four bonds (MAP4).^20^ The fingerprints were computed with the RDKit toolkit and the Python language.

Molecular complexity was assessed by CSP3, which corresponds to the fraction between sp^3^ hybridized carbon atoms and the total amount of carbon atoms, as an approximation of the degrees of freedom of molecules,^34^ and the DataWarrior complexity index. The latter index consists of the calculation of every different (unique) connected subgraph normalized over the molecular size. The more distinct the fragments are, the more complex the molecule is.^35^

### 2.5 Scaffold Content and Diversity

There are several methods to compute the molecular scaffolds of a molecule.^36^ In this study, we used the scaffold definition proposed by Bemis–Murcko, which is composed of the ring systems and linkers connecting them, and then all side chains were removed.^37^ Based on the scaffold content, we plotted the cumulative retrieval fraction of the database against the fraction of cyclic systems, the cyclic system retrieval (CSR) curve, which permits a direct comparison of the scaffold diversity of databases. The Shannon entropy of the most populated scaffolds was computed as a further estimation of the scaffold diversity.^38^ The scaled Shannon entropy is a metric that has values in the range of zero (minimum diversity, all compounds share the same scaffold) to one (maximum diversity: each compound has its unique scaffold).

### 2.6 Maximum Common Substructure Analysis

Taylor–Butina clustering was first applied to the databases based on their fingerprint representation according to ECFP4, using the algorithm described by Taylor in 1995,^39^ and later by Butina in 1999^40^ for unsupervised clustering. The maximum common substructure (MCS) of two compounds is the largest substructure that appears in both molecules. The MCS algorithm works by extracting the MCS (containing the most vertices and edges of the graph, atoms and bonds of the molecules, as possible) from two molecules represented as graphs.

ECFP4 1024-bits was implemented due to its robustness in terms of structural feature changes^41^ and previous successful reports of implementation in similarity approach applications related to medicinal chemistry.^42^ The Taylor–Butina clustering algorithm uses spheres of exclusion at a given Tanimoto similarity index level. The algorithm traces a centroid in each case and groups neighboring molecules belonging to a cluster that has an index above or equal to the predefined cutoff.

Compounds with MW higher than 1000 g/mol were disregarded to reduce the computational cost. The final data set consisted of 47,268 compounds, after disregarding the chirality and filtering the compounds by MW. The cutoff of the similarity index defined in the present work was 0.05, based on the inherent differences among acyclic compounds, relative to unsaturations and chirality (molecular complexity). Then, each generated cluster was represented in terms of its population of compounds. Finally, MCS was computed within each cluster.

### 2.7 Natural Product Likeness

The NPL score, developed by Ertl et al.,^43^ is a metric that measures how molecules are similar to the structural space covered by natural products and efficiently separates NPs from synthetic molecules. The NPL ranges between −5 (if the compound is more similar to a synthetic compound) and 5 (if the compound is more similar to a natural product). In this work, we computed the NPL score for FooDB, which contains food components, UNPD-A, a database containing the 14,994 structurally most diverse natural products from UNPD, and FDA-approved drugs.

### 2.8 Structural Diversity

The structural diversity of food components was analyzed through a comparison with FDA-approved drugs and natural product (UNPD-A) sets of compounds in terms of their distribution of similarity values computed with the Tanimoto coefficient using four molecular fingerprints: MACCS Keys (166-bits),^18^ ECFP^19^ of 1024 bits with a diameter of 4 (ECFP4) and 6 (ECFP6), and MAP4.^20^ For food components, five random samples of 1000 compounds each were extracted, and calculations were carried out. It has been demonstrated that multiple random sampling of 1000 compounds from large data sets is a valid approach to quantify the entire database pairwise fingerprint-based diversity.^44^

### 2.9 Chemical Multiverse Visualization

The three databases, FooDB, UNPD-A, and FDA-approved drugs, along with FooDB commercially available, were compared in their multiverse visualizations through different sets of molecular descriptors. A chemical multiverse is a group of “alternative” chemical spaces of a set of compounds defined by a distinct set of molecular descriptors. Each chemical space is an M-dimensional Cartesian space, and each dimension represents the descriptors or features encoding a molecule. The length of descriptor sets defines the number of dimensions of each chemical space.^21^ Dimensionality reduction for chemical space visualization was achieved using t-distributed stochastic neighbor embedding (t-SNE) according to the bits-based fingerprints previously computed (vide supra). t-SNE is a nonlinear method, which uses t-distribution instead of the linear one used in PCA. This approach allows t-SNE to display a wider distribution of points along the graph, preserving the local structure, or clustering of the data.^45^

### 2.10 Commercially Available Chemical Space of Food Components

The number of compounds in FooDB that are commercially available was estimated through a comparison with the commercial compounds listed in ZINC20.^27^ The data set of commercially available food components, including both in-stock and made-on-demand components, was obtained from a ZINC20 query using canonical SMILES in FooDB, retaining and disregarding the chirality of structures.

### 2.11 Chemical Profiling and Classification

Classification of compounds by their structural family was achieved using NPClassifier, a neural-network-based approach that automatically classifies natural compounds according to their pathway, superclass, and class. At the time of writing (August 2023), NPClassifier includes seven pathways based on biosynthetic routes, such as polyketides, amino acids and peptides, fatty acids, shikimates, alkaloids, carbohydrates, and terpenoids. Each pathway is categorized into 70 superclasses from general categories of metabolites and general molecular shapes. Finally, superclasses are subdivided into 672 classes that represent specific compound families, functional groups, or scaffold clusters within a superclass.^46^ NPClassifier has been used to previsualize the biosynthetic pathway of compounds in data sets of natural products with medicinal interest, such as the IMPPAT (Indian Medicinal Plants, Phytochemistry And Therapeutics) 2.0 database.^47^ It has also been used to link biosynthetic pathways of numerous metabolic products with their corresponding biosynthetic gene clusters as an approximation to associate the fields of metabolomics and genomics.^48^

## 3 Results and Discussion

### 3.1 Data Sets

Table 1 summarizes the number of compounds of the three data sets studied in this work and the subset of commercially available compounds from FooDB. Table 1 also summarizes the results of the molecular similarity computed based on molecular fingerprints (MACCS keys, ECFP4, ECFP6, and MAP4), structural complexity quantified based on CSP3, and natural-product likeness estimated with the NPL score, as described in the Methods section. For FooDB and its purchasable subset, the number of curated compounds considering chirality information and disregarding chirality are included.

**Table 1:** Summary of the Structural Diversity, Complexity, and NPL Score of the Food Components and Reference Data Sets

data set	size (initial)	size (curated)	mean (median) similarity				mean CSP3^c^	NPL score^c^
			MACCS keys-167 bits	ECFP4 1024 bits	ECFP6 1024 bits	MAP4 2048 bits		
FooDB	70,477	68,658 (chiral SMILES); 52,856 (nonchiral SMILES)	0.65 (0.62)	0.44 (0.47)	0.40 (0.42)	0.23 (0.20)	0.76	0.67
UNPD-A^a^	14,994	14,994	0.35 (0.34)	0.10 (0.09)	0.08 (0.08)	0.01 (0.00)	0.52	1.51
FDA	2587	2324	0.30 (0.30)	0.10 (0.09)	0.08 (0.08)	0.01 (0.00)	0.45	0.02
FooDB purchasable^b^		3330 (chiral SMILES); 2422 (nonchiral SMILES)	0.26 (0.22)	0.12 (0.09)	0.09 (0.08)	0.02 (0.00)	0.45	0.51

a Data set curated from the source.^25^

b Subset extracted from our previously curated version of FooDB.

c Computed using nonchiral SMILES.

### 3.2 Data Sets Overlap

**Figure 1:**
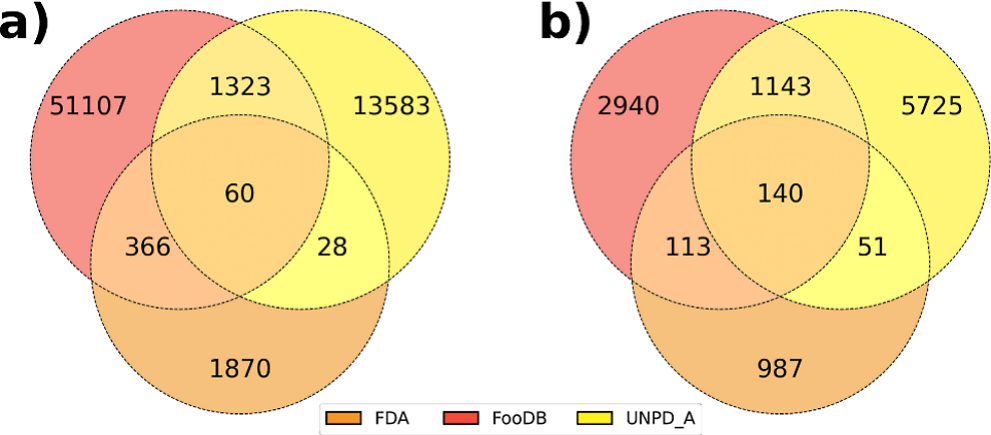
Unique and overlapping structures between FooDB (red), natural products in UNPD-A (yellow), and FDA-approved drug databases (orange). Structural content was analyzed regarding (a) entire compounds and (b) molecular scaffolds.

Figure 1 shows the overlap among the three primary databases in terms of their compounds (Figure 1a) and molecular scaffolds (Figure 1b). According to the results in Figure 1a, there are 66,560 unique compounds among the three data sets. The most significant overlap is between natural products (UNPD-A) and FooDB, with 1383 compounds, of which 60 are also shared with approved drugs (available data on GitHub at https://github.com/DIFACQUIM/Food_chemicals_characterization). FDA-approved drugs shared 88 compounds with natural products (UNPD-A) and 426 with FooDB. Overlapping between all the three data sets is little (2.6%), and 97.4% of the total compounds are unique, belonging to a single set. Breaking down each data set, 90.6% of natural products (UNPD-A), 96.7% of FooDB, and 80.5% of FDA-approved drugs belong to only a single set. It is remarkable that even when this study is being carried out with a subset of natural products with a considerably smaller size than FooDB, 1383 compounds in FooDB are also present in the UNPD-A. Then, we could expect that this overlap would be larger with a database as massive as all UNPD compounds^26^ or the Collection of Open Natural ProdUcTs, COCONUT,^49^ maintaining the observed pattern of previous studies.^16^

**Figure 2:**
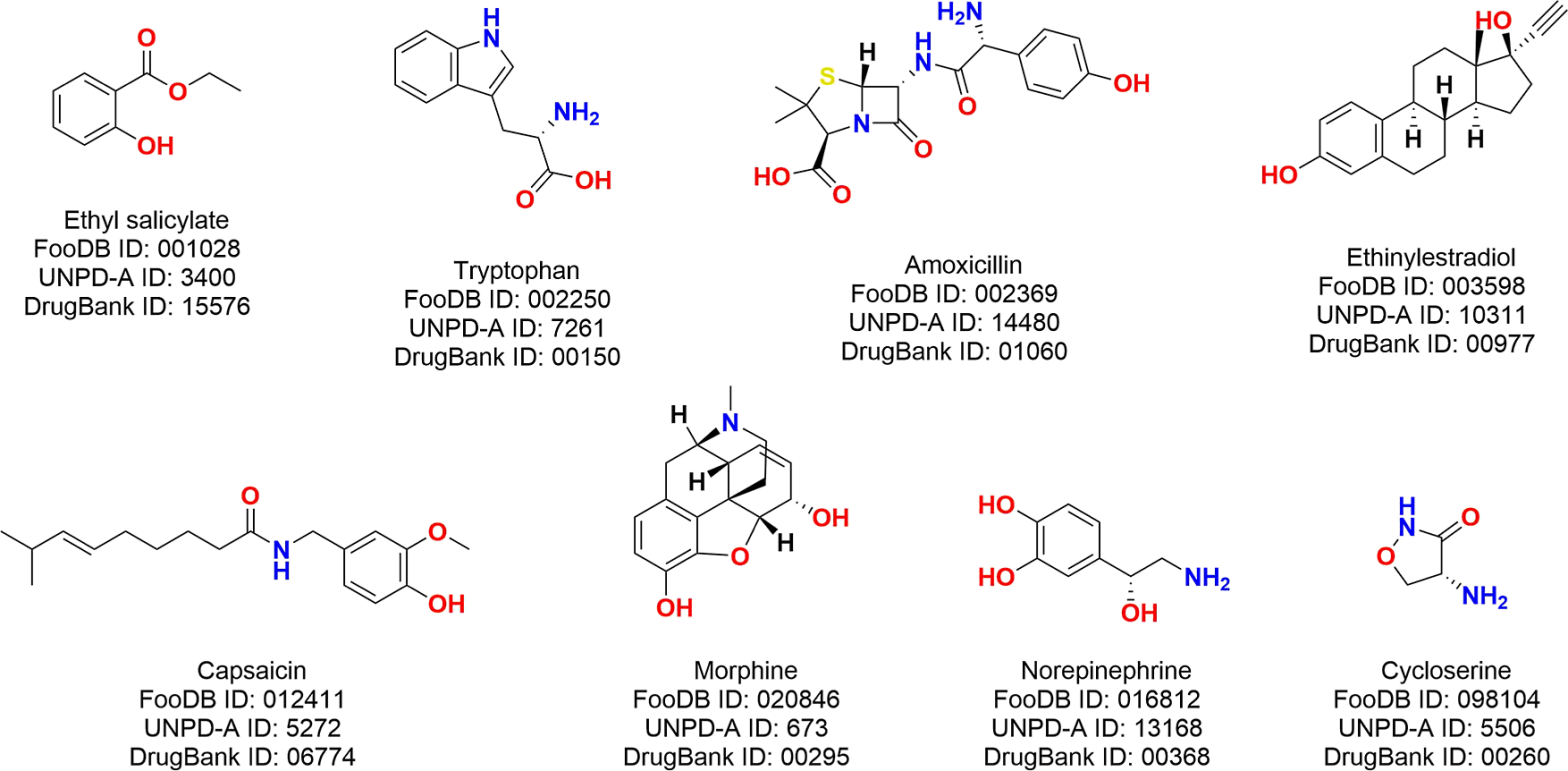
Examples of compounds that are categorized as food components, natural products, and FDA-approved drugs. Chirality is added after pairing the databases according to the FDA-approved specifications. The compound identifiers (IDs) in each database is shown.

Among the 60 common compounds in the three principal databases, it is not surprising to find food components (nutrients and additives), which are used in the clinic to treat nutrient deficiencies, the reason why they are present in FDA-approved drugs (Figure 2). Examples are nicotinamide, adenine, succinic acid, or cystine, which can be found in alimentary supplements.^50^d-glucose and amino acids such as d-serine, l-glutamine, and tryptophan for parenteral nutrition were also detected. The latter has also been used as a treatment for patients with depression.^51^

There is also the presence of drugs from natural products, such as morphine, for severe and chronic pain management and a precursor of semisynthetic opioids. Ethyl and methyl salicylate are common predecessors of the widely used acetylsalicylic acid, such as the NSAID naproxen. Among antibiotics, there is amoxicillin, a semisynthetic derivative of penicillin, which can be found as a milk contaminant,^52^ also, erythromycin among macrolides and cycloserine, a d-alanine analogue. It is also remarkable that the presence of ethinylestradiol, an estradiol derivative for oral contraceptive formulation, FDA-approved since 1943, present in almonds, apples, peaches, and coffee.^53^ Additionally, digitoxin, an approved drug for cardiac affections, belongs to the family of cardiac glucosides or cardiotonics and is found in *Digitalis purpurea* extract.^54^ Capsaicin, a compound in the Mexican diet, is also a topic anesthetic for neuropathic pain.^55^ Norepinephrine as a catechol is widely present in natural products, citrus, and vegetables such as potatoes.^56^

Regarding the scaffold content, 11,099 structures were identified, of which 140 (1.3%) were common to the three databases. The number of unique scaffolds was 9652 (87.0%), taking into account all the studied compounds at once. This translates to 67.8% of unique scaffolds from FooDB, 81.1% from natural products (UNPD-A), and 76.5% from FDA-approved drugs. It is notable that despite FooDB being larger than UNPD-A, and so on FDA-approved drugs, the latter present a larger percentage of different unique scaffolds. This is related to the high fraction of the database with acyclic structure, as described and discussed in Sections 3.3–3.5.

### 3.3 Distribution of Physicochemical Properties and Constitutional Descriptors

**Figure 3:**
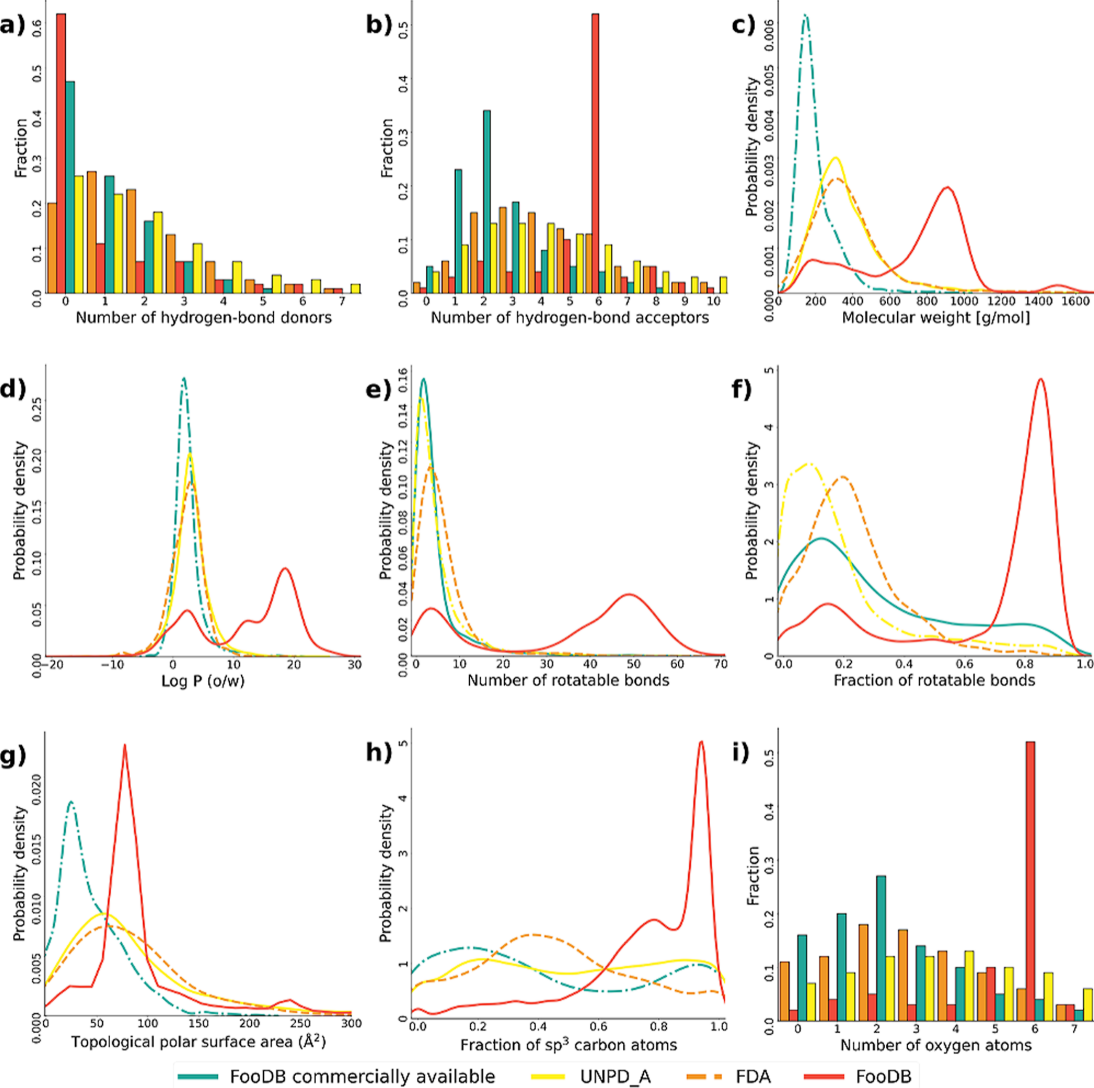
Distribution of physicochemical properties and constitutional descriptors of interest among FDA-approved drugs (orange), compounds from FooDB (red), commercially available compounds from FooDB (green), and natural products (UNPD-A; yellow): (a) number of hydrogen-bond donors, (b) number of hydrogen-bond acceptors, (c) MW, (d) logP, (e) number of RB, (f) fraction of RB, (g) TPSA, (h) fraction of sp^3^ carbon atoms, and (i) number of oxygen atoms. Dotted lines are used for the ease of visualization.

Figure 3 shows the distribution of physicochemical properties and constitutional descriptors of interest in the four databases, as histograms, representing the fraction of the total amount of compounds in each case or as a probability distribution, which represents the occurrence of different values along the variable. Regarding properties related to drugs of oral administration, as described by Lipinski^57^ and Veber,^58^ it is notable that food components had 1.59 HBD on average (median: 0.00; standard deviation: 3.5), natural products had 2.51 (median: 2.00; standard deviation: 3.2), and approved drugs had 2.44 (median: 2.00; standard deviation: 3.7). Referring to the HBA, food components had 6.75 on average (median: 6.00; standard deviation: 4.9), natural products had 5.58 (median: 4.00; standard deviation: 5.0), and FDA-approved drugs had 5.29 (median: 4.00; standard deviation: 4.6). With respect to the number of heteroatoms per molecule, food components had 7.39 on average (median: 6.00; standard deviation: 5.8), and approved drugs had 7.50 (median: 6.00; standard deviation: 7.0), while natural products had 6.02 (median: 5.00; standard deviation: 5.1).

In terms of molecular size, the average MW was 736.41 for food components (median: 821.37; standard deviation: 326.9), 371.94 for natural products (median: 330.29; standard deviation: 196.4), and 387.38 for approved drugs (median: 337.37; standard deviation: 272.0), demonstrating a tendency for larger sizes of food components. According to polarity as measured with logP and TPSA, food components have average values of 12.22 (median: 14.87; standard deviation: 8.1) and 103.53 (median: 78.90; standard deviation: 90.3), natural products ^OBJ^2.94 (median: 2.87; standard deviation: 3.0) and 90.78 (median: 69.67; standard deviation: 82.7), and approved drugs 2.27 (median: 2.55; standard deviation: 2.9) and 95.73 (median: 74.60; standard deviation: 106.4), respectively.

For the fraction of RB, food components have an average of 0.65 (median: 0.80; standard deviation: 0.3), natural products 0.19 (median: 0.13; standard deviation: 0.2), and FDA-approved drugs 0.23 (median: 0.20; standard deviation: 0.2). This is in accordance with the content of aromatic rings, for which food compounds present 0.32 on average (median: 0.00; standard deviation: 0.9), natural products 1.28 (median: 1.00; standard deviation: 1.5), and approved drugs 1.54 (median: 1.00; standard deviation: 1.3). This result is also related to the content of scaffolds analyzed in Section 3.4, showing a smaller abundance of rigid substructures in the food components.

In general, food component properties have had a long variation between previous versions of FooDB and the current updated version. For example, the average content of HBD decreased from 3.5 to 1.59 and HBA from 7.2 to 6.75. The CSP3 average content changed from 0.62 to 0.76 (median: 0.82; standard deviation: 0.2), remarking an increasing presence of acyclic apolar substituents, which is supported by the variation in logP average from 4 to 12.22 and TPSA average from 124.7 to 103.53. The average MW increased from 490 to 736.41, and the number of RB increased from 13.6 to 35.25, which allows us to argue for a large presence of long aliphatic chains. These observations are supported by the constitutional descriptors (see Table S1 and Figure S1 in the Supporting Information), showing a larger number of acyclic structures in food components than in natural products and approved drugs.

The high content of oxygen atoms, along with the low tendency of polarity among food compounds, is explained by the abundance of fatty acids, and the scarce presence of cyclic structures and scaffold contents also supports it. These results are in agreement with chemical classification, as described in Section 3.9.

The results found in the present study, which includes a 3 times larger set of food components in front of previous versions, showed similar trends as previous reports of descriptor analysis for FDA-approved drugs and different collections of natural products.^59,60^

The distribution of all calculated properties has shown a broader range for food components than natural products and approved drugs, in accordance with the large structural diversity of food components. The distribution of properties further shows a distinct balance with a tendency toward low polarity, large size, and high structural complexity as measured with CSP3. It is also remarkable that the purchasable subset of compounds from FooDB presents a narrow distribution, showing that the commercial availability of food-source compounds is still limited and focused on molecules with similar properties to natural products and approved drugs. Complete statistical results are in Table S1.

#### 3.3.1 Structural Complexity

The content of chiral centers and CSP3 are widely employed approximated metrics to quantify molecular complexity. We found that the average CSP3 was 0.79 (median: 0.89; standard deviation: 0.2) for food components, in contrast with 0.52 (median: 0.52; standard deviation: 0.3), for natural products and 0.45 (median: 0.43; standard deviation: 0.3) for FDA-approved drugs (Figure 3 and Table 1). The average content of chiral centers in food components structures was 2.73 (median: 1.00; standard deviation: 4.9), while natural products had 3.81 (median: 2.00; standard deviation: 5.1), and approved drugs were 2.30 (median: 1.00; standard deviation: 3.8). These results indicate that in terms of three-dimensionality disposition, food components in FooDB are highly complex, and according to the presence of stereochemical centers, their complexity is between natural products and approved drugs, dropping since the previous characterization.^11,61^

**Figure 4:**
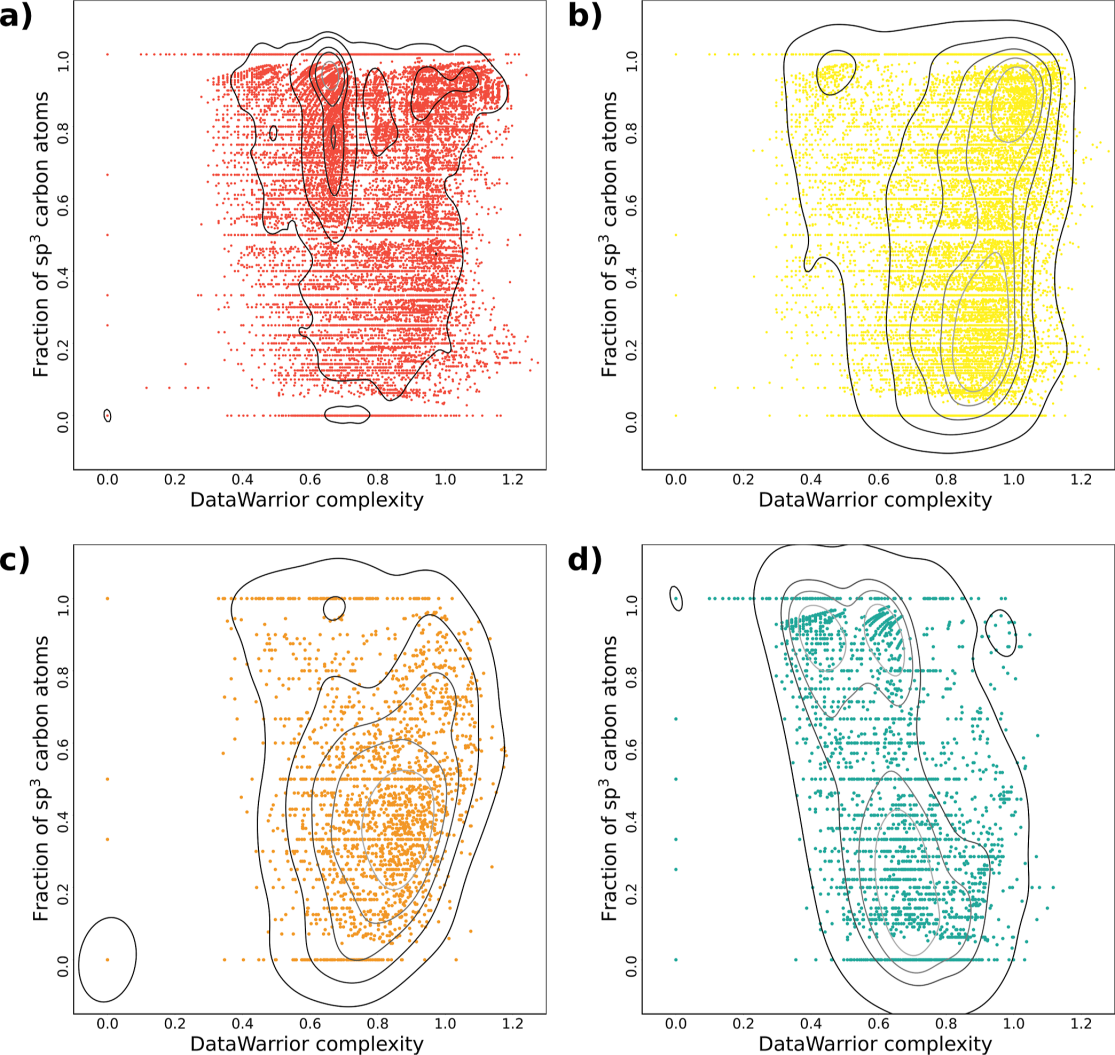
Density plot of pairwise comparison between CSP3 and DataWarrior complexity index pairwise comparison computed for (a) food components (FooDB), (b) natural products (UNPD-A), (c) FDA-approved drugs and (d) commercially available compounds from FooDB. Surfaces circle regions with a high density of data points.

In addition to CSP3 and chiral centers, there are other metrics to quantify structural complexity, as described in detail elsewhere.^61^Figure 4 shows the relationship between the CSP3 fraction and DataWarrior complexity index (described in the Methods section) for the studied data sets, with points representing chemical compounds. Regions with a high density of data points can be interpreted as tendencies in chemical complexity of the database and, by approximation, the kind of compounds they represent. Food components cover all spectra of CSP3 complexity, but 75% of them are sharply located in values higher than 0.712 and a mean of 0.792 (median: 0.89; standard deviation: 0.2). Natural products (UNPD-A) and FDA-approved drugs had a mean CSP3 of 0.519 (median: 0.52; standard deviation: 0.3) and 0.454 (median: 0.43; standard deviation: 0.3), respectively, and broader distributions (50% of natural products were from 0.250 to 0.800 and for approved drugs from 0.263 to 0.632) (Table S2). FDA-approved drugs generally have compounds with fewer chiral centers^62^ so that they are more synthetically accessible.^63^ In terms of the DataWarrior complexity index, food components had a mean of 0.697 (median: 0.67; standard deviation: 0.1), and 75% of compounds were higher than 0.654. In contrast, both natural products and approved drugs had higher DataWarrior complexity. UNPD-A had a mean of 0.832 (median: 0.87; standard deviation: 0.2), and FDA-approved drugs had a mean of 0.780 (median: 0.81; standard deviation: 0.2). In addition, 75% of compounds of UNPD-A had a DataWarrior complexity index of more than 0.728 and FDA-approved drugs of more than 0.681 (see Figure S2). This can be related to the presence of macronutrients in food components with more symmetric acyclic structures. At the same time, natural products and FDA-approved drugs are richer in scaffolds that contribute to unique fragment count. This is consistent with previous complexity characterizations of natural products and drug-type sets of compounds.^64^

Molecular complexity is widely interpreted as an indicator of selectivity regarding biological interaction, among small-molecule libraries.^65^ Food components, as well as natural products, come from natural sources, which have developed millennial mechanisms of biosynthesis, involving elaborated systems that had evolved for millions of years, producing molecules that had been in contact with mammals for thousands of years. Those processes give food chemicals (nutrients) the quality of being building blocks to sustain life; at the same time, they are sources of bioactive molecules as secondary metabolites.^66^

### 3.4 Scaffold Content

**Figure 5:**
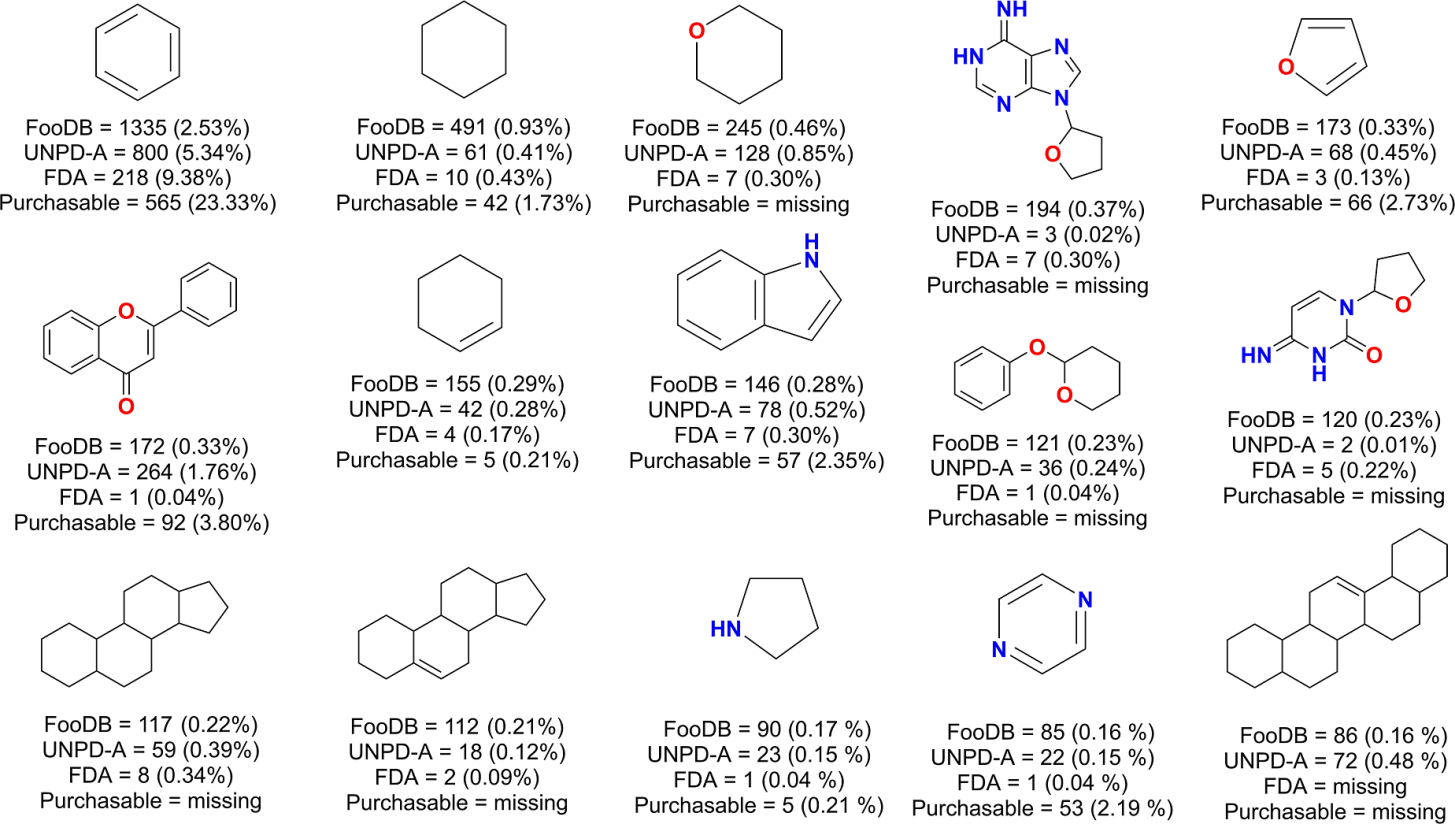
Fifteen most frequent scaffolds from FooDB and their presence in the four data sets. Additionally, 38,330 (72.52%) of FooDB, 1743 (11.62%) of UNPD-A, 265 (11.40%) approved drugs, and 821 (33.90%) commercially available FooDB are acyclic compounds.

Figure 5 shows the frequencies of the most common scaffolds in the four data sets. Food components show a clear tendency to the content of acyclic compounds, with 38,330 (72.52%) compounds, followed by single ring compounds, both aromatic (2.53%), saturated (0.93%), and heterocyclic saturated tetrahydropyran type (0.46%). Benzene remains the most common scaffold, as in the previous analysis of the database,^11^ following the trend of most small-molecule databases, especially of natural origin.^37,59,67^ FooDB had 4336 different scaffolds, of which a small amount (306–7%—scaffolds) were present in commercial catalogs. UNPD-A natural products had 7059 different scaffolds and FDA-approved drugs 1291.

The Shannon’s entropy for each data set was computed for the 15 most frequent scaffolds to quantify and compare their scaffold diversity. The scaled Shannon’s entropy for natural products (UNPD-A) was 0.67, for food components was 0.18, FDA-approved drugs had 0.63, and purchasable food components had 0.65. Since UNPD-A is a database designed to be as diverse as possible, it is not unexpected to be the most diverse of the studied databases, in terms of scaffolds. FDA-approved drugs, as well as purchasable food components, are small groups of highly screened compounds due to their functional-focused generation, while for food components, several compounds are distributed in a relatively low number of chemotypes.

**Figure 6:**
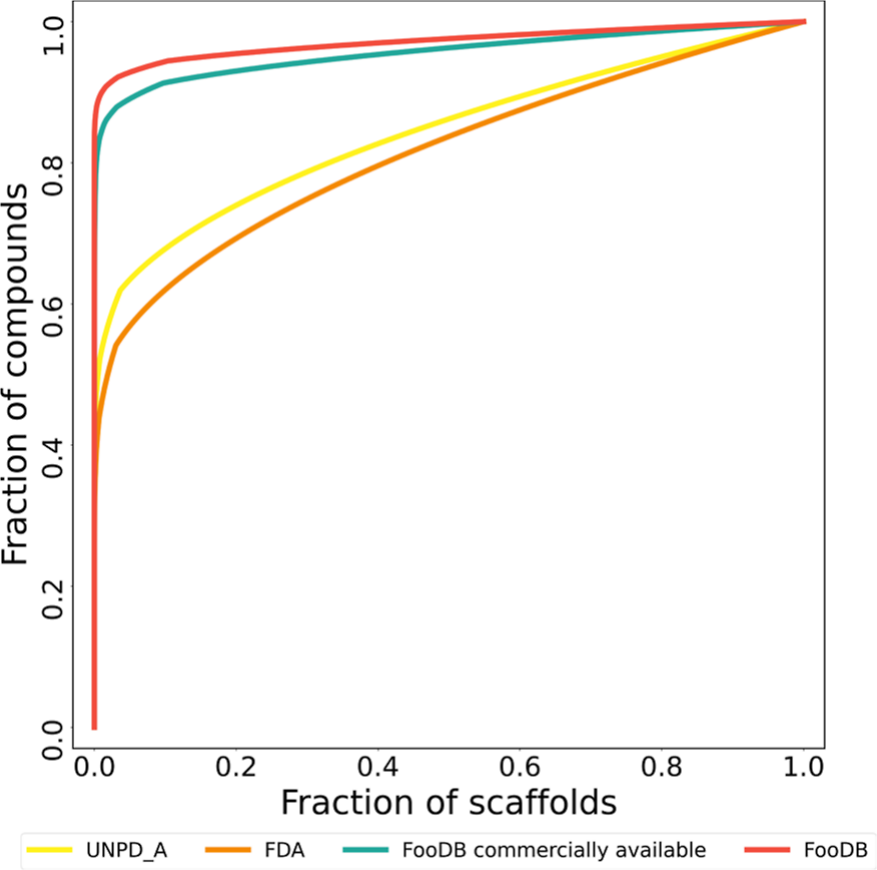
CSR curves for natural products (UNPD-A, yellow), FDA-approved drugs (orange), commercially available compounds of FooDB (green), and compounds of FooDB (red).

Figure 6 shows a CSR curve, which consists of the representation of the cumulated fraction of compounds covered by the total distribution of scaffolds, giving rise to a direct comparison between abundance and scaffold diversity. By using it, the rapid accumulation of compounds from food components is evident, along with a low fraction of present scaffolds. According to this approximation, natural products are the most scaffold-diverse database, followed by FDA-approved drugs, and commercially available food components in third place.

It is remarkable that the 15 most frequent scaffolds in FooDB, represented by 3642 compounds (6.9% of FooDB), cover 298 (12.73%) compounds of FDA-approved drugs, and this number increases to 563 (24.13%) considering the acyclic systems. This trend is almost conservative according to the previous characterization of FooDB.^11^

The high proportion of acyclic compounds in food components was already noticed in the previous version of FooDB with fewer compounds.^11^ These results highlight the potential of food components to discover new bioactive molecules, develop nutraceuticals, and also point out the large diversity of sources and biosynthetic pathways in nature that produce food chemicals. Also, the high proportion of acyclic compounds is a distinctive feature of food components.^17^

### 3.5 Maximum Common Substructure Analysis

**Figure 7:**
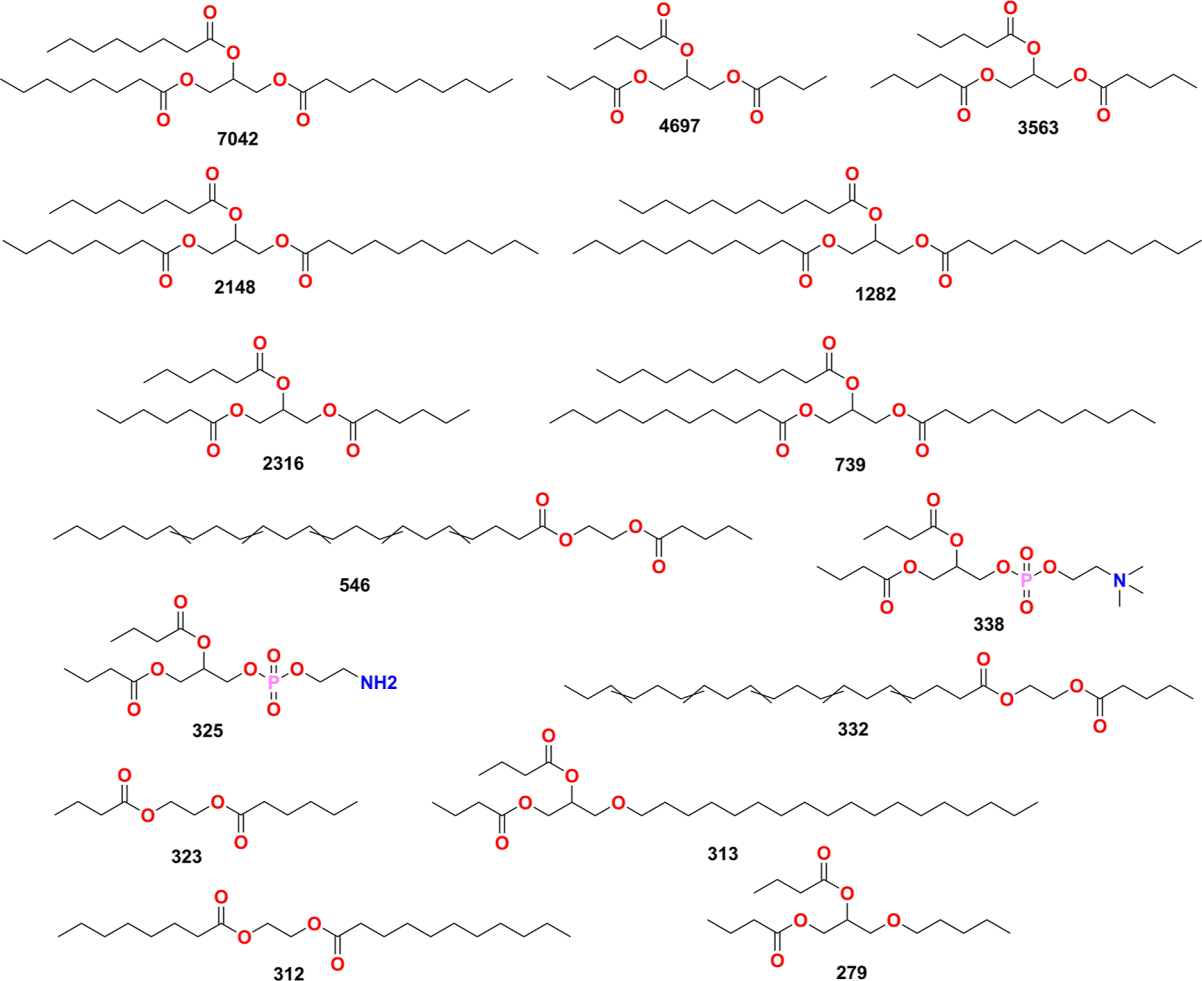
Representative maximum substructures of food components in FooDB computed for some clusters. The number below each structure is the number of molecules that share a substructure within each cluster.

As described in the Methods section, the MCS of food components present in FooDB was computed starting with a nonhierarchical clustering based on the Taylor–Butina classical algorithm of the compounds based on their ECFP4 representation. The dissimilarity threshold employed was fixed at 0.05 to guarantee the convergence of molecules to a common substructure that can be achieved by the MCS algorithm. Figure 7 shows a consolidation of the first 15 MCS for food components.

In concordance with previous analyses, most of the MCS corresponds to triacylglycerides.^11,14^ Acyclic molecules are of high complexity due to the rotatability of their bonds. Figure S3 shows examples of compounds present in different computed clusters. This approach was also applied to natural products and FDA-approved drugs; however, significant clustering and substructure searching were not achieved at the threshold fixed. Figure S4 shows the main substructures computed for natural products and FDA-approved drugs.

As shown in Figure S3, the compounds grouped in the first clusters for food components belong to different classes of lipids, mostly triacylglycerides. Following the results of the chemical descriptors’ analysis, these findings distinguish along the high occurrence of acyclic molecules (see Section 3.4) and the chemical profiling of food components (see Section 3.9).

### 3.6 Natural Product Likeness

**Figure 8:**
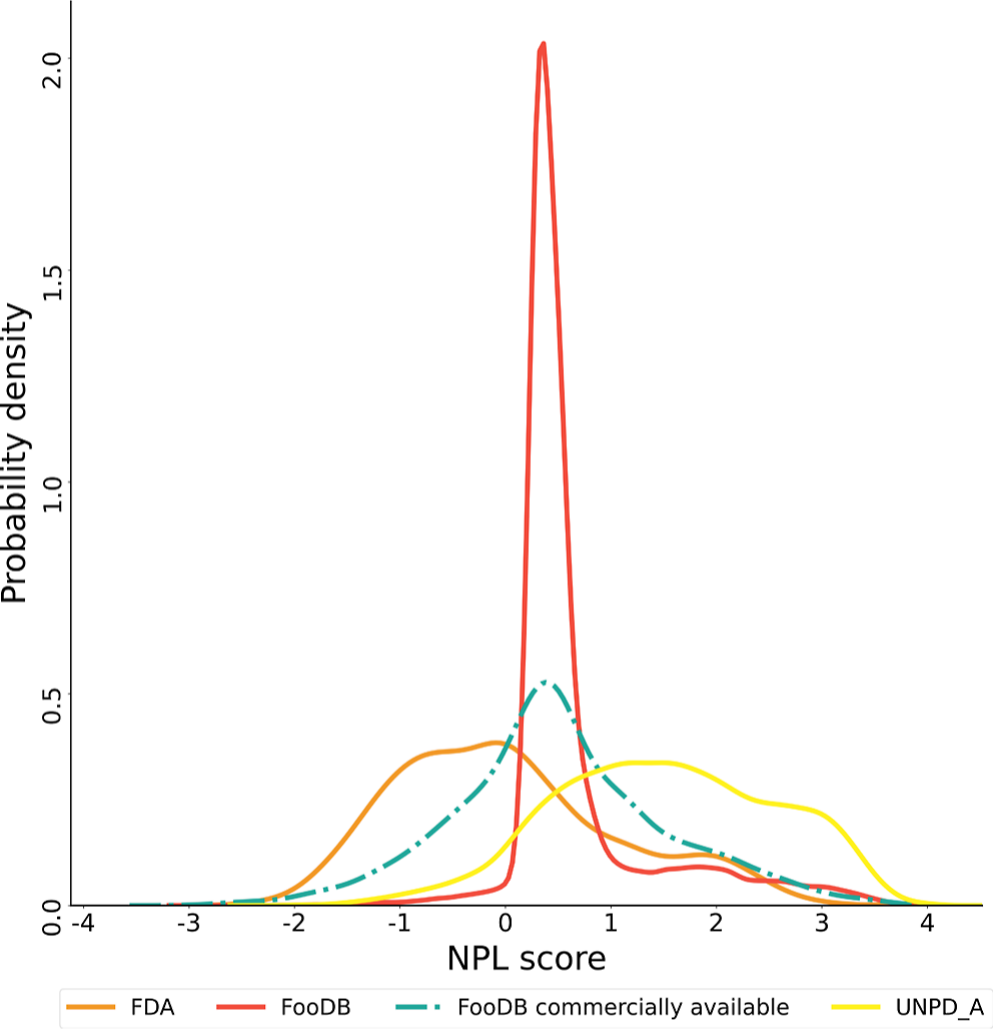
Distribution of probability density of the NPL score among approved drugs (orange), compounds of FooDB (red), commercially available compounds of FooDB (green), and natural products (UNPD-A; yellow). Dotted lines are used for ease of visualization.

Table S3 summarizes the NPL scores calculated for food components in FooDB (mean = 0.67, median = 0.44, and standard deviation = 0.71), natural products from UNPD-A (mean = 1.51, median = 1.51, and standard deviation = 1.05), FDA-approved drugs (mean = 0.02, median = −0.10, and standard deviation = 1.08), and the FooDB commercially available compounds’ subset (mean = 0.51, median = 0.44, and standard deviation = 1.02). As a reference, only 25% of natural products in UNPD-A drop partially into negative values (min = −2.14 and Q1 = 0.75), while more than 75% of food components from FooDB drop into values below these referential values (min = −2.96 and Q3 = 0.64), showing approximate behavior to FDA-approved drugs in this respect (min = −2.50 and Q3 = 0.63). Studying the distribution of NPL scores for the four data sets evidenced more clearly that most food compounds fall into the range between −1 and 1 (Figure 8), while approved drugs tend to have negative values, associated with synthetic origin,^43^ and natural products had NPL scores closer to 5, in accordance to the previous description of the data set.^25^

### 3.7 Fingerprint-Based Structural Diversity

**Figure 9:**
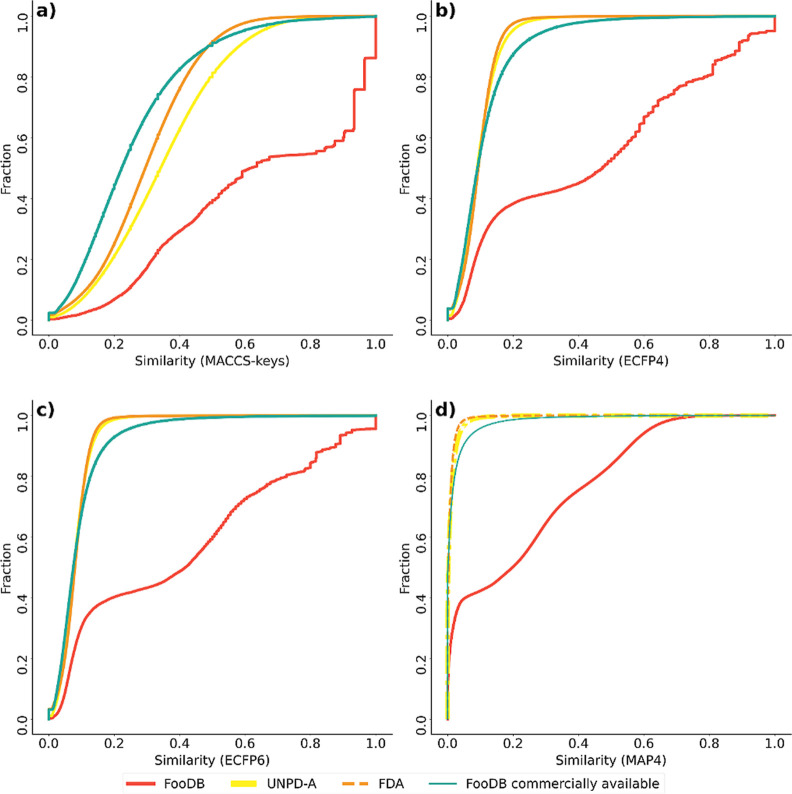
Cumulative distribution functions of the pairwise Tanimoto similarity using (a) MACCS keys (166-bits), (b) ECFP4, (c) ECFP6, and (d) MAP4 as molecular representations. Approved drugs (orange), compounds of FooDB (red), commercially available compounds of FooDB (green), and natural products (UNPD-A; yellow). Dotted lines are used for ease of visualization.

Based on structural fingerprints, we compared the diversity of food components with the reference databases of natural products and FDA-approved drugs (vide supra). To this end, we used the Tanimoto similarity index with structural fingerprints of different designs, as detailed in Methods. Figure 9 shows the cumulative distribution function of the pairwise similarity values calculated with MACCS keys (166 bits), ECFP4, and ECFP6 (1024 bits) and the recently developed MAP4 (2048-bits). Results indicate that independent of the fingerprint representation, food components had the least molecular diversity, which can be seen in the CDF, and in greater values for mean and median similarity (Table S4 in the Supporting Information). The relatively low fingerprint-based diversity is associated, at least in part, with the high proportion of structurally similar acyclic compounds (70%) and a sudden increase in the CSR curve (Figure 9). The low fingerprint diversity is also related to the profile of CSP3, fraction of RB, number of oxygen atoms, and ring system content, which have narrow distributions (the latter also impact the distribution of some physicochemical properties such as TPSA, and logP to some extent; see Section 3.3). These results differ from the previously reported diversity analysis of food components^11,16^ but can be related to the enrichment of the database with acyclic compounds in the last years, such as triacylglycerols, as is discussed in Section 3.9. In contrast, natural products, as well as FDA-approved drugs and commercially available food components, presented a higher structural diversity and lower mean and median values of pairwise similarity. Those results are related to a larger scaffold diversity, a broad distribution of physicochemical properties, and constitutional descriptors. Results of computed similarity coefficients for UNPD-A are comparable to those of studies published before, which validates the chemoinformatics methodology used.^25^

Commercially available food components had a CDF and descriptive statistics of similarity distribution, close to FDA-approved drugs and natural products, especially with ECFP and MAP4 fingerprints (Table S4). For MACCS keys, the quality of the food compound subset is undetectable by comparison, showing even higher fingerprint-based diversity than reference data sets.

As detailed in Methods, we computed fingerprints of different designs, which can be divided into two major groups: molecular dependent (MACCS keys) and molecular independent (ECFP and MAP4).^68^ The fingerprint design is associated with the degree of complexity and specificity (or structural resolution) that a fingerprint can capture. MACCS keys describe a generic set of 166 structural features, quantified as if it is present or absent.^18^ In contrast, ECFP and MAP4 describe specific connectivity features of each molecule along a user-defined radius, giving them higher variability and specificity. Therefore, pairwise similarity values calculated for the same sets of compounds with higher-resolution fingerprints (such as ECFP and MAP4) are lower than those computed with a low-resolution fingerprint.^19,20^ Such dependence on the magnitude of the pairwise similarity values with fingerprints of different designs has been noted in previous studies comparing different compound databases.^69^ These trends are evident in this study for all data set similarity distribution statistics (mean and median, see Table S4 in the Supporting Information) which show a decreasing tendency while fingerprint complexity and specificity increase.

### 3.8 Chemical Multiverse Visualization

**Figure 10:**
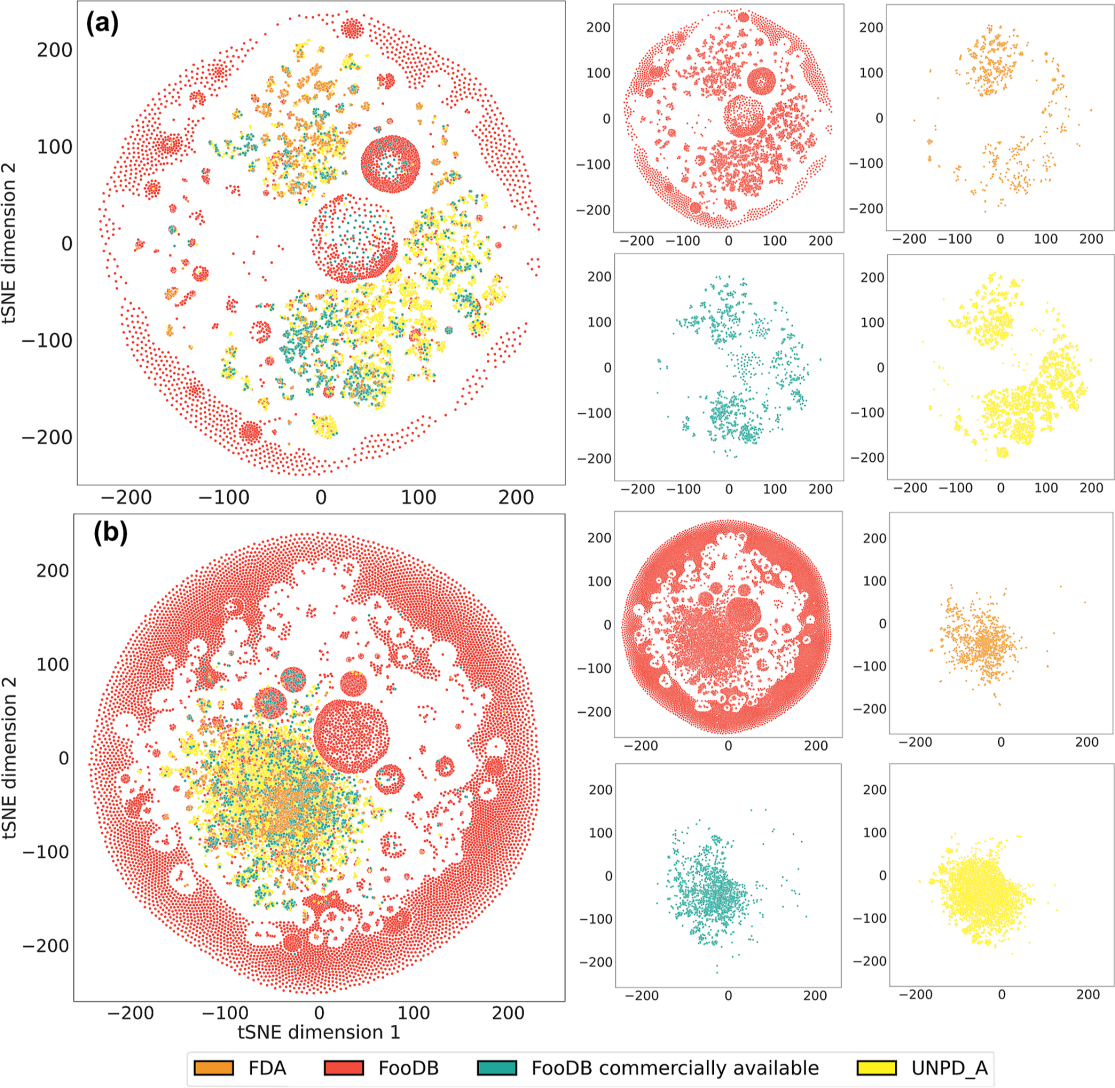
Chemical multiverse visualization of food components and their comparison with natural products and approved drugs using t-SNE as dimensionality reduction and (a) MACCS keys (166-bits) and (b) ECFP4 (1024-bits) as molecular representations. On the left are superimposed databases and on the right are individual databases multiverse representations.

A chemical multiverse is a set of distinct chemical spaces, each defined by a different group of descriptors. The advantage of the chemical multiverse concept over individual chemical spaces is that the former leads to more comprehensive information related to compound relationships.^21^Figure 10 shows a chemical multiverse of the compound data sets obtained with MACCS keys (166-bits) (Figure 10a) and ECFP4 (1024-bits) (Figure 10b) fingerprints, employing t-SNE as a visualization method. Results on the visualization of the chemical multiverse are in agreement with molecular diversity. Using MACCS keys as molecular representation, food components approximately covered the chemical space of natural products as well as approved drugs. This finding can be associated with the sharing of chemotypes among molecules in the three databases, regarding the nature of the molecular independent fingerprint. The wide spread of food components by MACCS key representation is low compared to ECFP4 and ECFP6 representation (see Figure S5) due to the large proportion of aliphatic structures and lack of scaffold diversity together with the low accuracy of the fingerprint by definition.^70^ However, there is a clear differentiation of compounds by region according to the database from which they come from. In contrast, both ECFP4 and ECFP6 display a better differentiation of food components among themselves because of the design of the fingerprint which privileges local diversity, instead of substructural coincidence, which increases their accuracy.^19^ However, ECFP4 representation visualization retained approximately the characteristic of pulling apart compounds by the original data set.

Despite the marked diversity of food components compared to natural products and approved drugs, it is remarkable that the chemical space of food components had a partial overlap with reference databases, which suggests that food components are a promising source of distinct and bioactive structures.

### 3.9 Chemical Profiling and Classification

**Figure 11:**
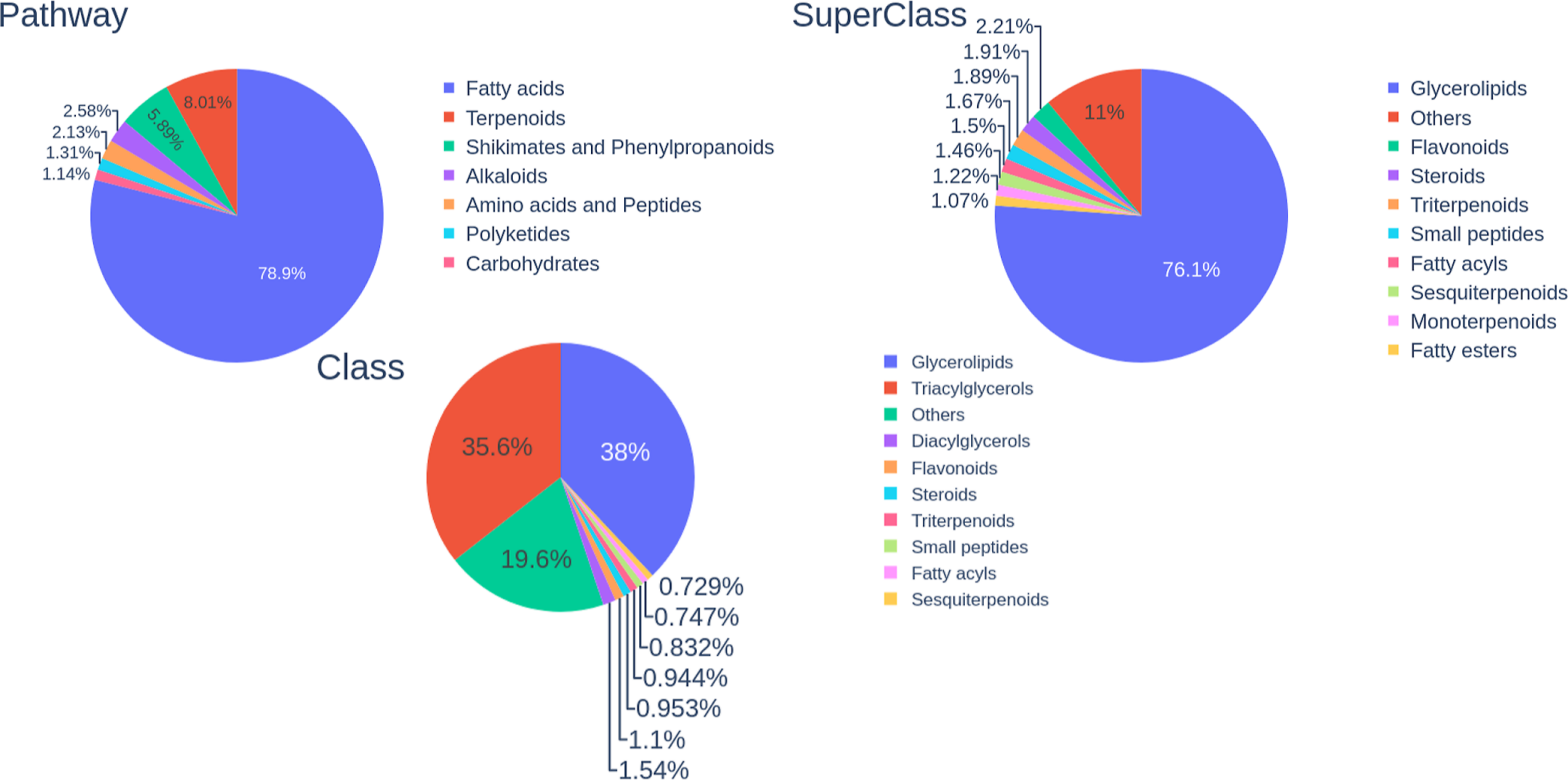
Chemical classification profiling predicted for food components (FooDB) according to their (a) biosynthetic pathway, (b) superclass, and (c) class. The classification was done with NPClassifier.

Using the NPClassifier neural network, we classified food components, natural products, and FDA-approved drugs, as described in the Methods section. This analysis (summarized in Table S5) confirmed that food components from FooDB mainly are fatty acids of the triacylglycerol class (Figure 11). Triacylglycerols are formed by a glycerol molecule, substituted by three aliphatic acyl groups, named fatty acid. These results agree with the characteristics previously described, such as long chain size, high MW, low polarity, and high CSP3.^71^ More than a thousand known fatty acids can act as substituents of glycerol, which give rise to a large number of molecules without necessarily a high structural diversity. Asymmetric triacylglycerols are common and present a stereocenter at C2 of glycerol, which explains the drop in the number of compounds across the chirality discard.^71^

Triacylglycerols play an important role in the human diet as they are the main lipidic component of foods and also commercial oils and fats.^71^ Current lipid studies are mainly guided to the development of functional foods and find replacing lipids that improve the nutritional contribution of foods.^72^ Despite the crucial role of lipids as components of metabolism, regulatory molecules, and energy stocks, their presence in FDA-approved drugs was 4.8% as fatty acids and less than 1% as glycerolipids such as triacylglycerols (see Table S7). However, commercially exploited food components showed good representativity of the set of FooDB, as their four biggest superclasses belong to the pathway of fatty acids, instead of not being triacylglycerols (see Table S8).

Natural products had a diverse class distribution; some of them represented the most abundant classes of FDA-approved drugs, related to alkaloids, the largest biopath in FDA-approved drugs (see Table S6).

### 3.10 Commercially Available Chemical Space of Food Components

While the search retaining chirality retrieved a total of 3330 compounds, ignoring chirality, the search gave a recovery of 2422 compounds. Although being a subset of food components in general, commercially available compounds from FooDB had contrasting, and even contrary, features, in terms of physicochemical and constitutional descriptors, scaffold content and CSR, NPL, chemical space, and multiverse coverage according to structural fingerprints, as well as similarity pairwise coefficient, using different fingerprint representations. Finally, chemical classification using NPClassifier showed that the most commercially exploited source of molecules is lipids and fatty acids, although with different compositions of the general set of food components.

## 4 Conclusions

Computational characterization of food components is of growing importance to develop further food components, not only for drug discovery and health-related benefits (e.g., nutraceuticals) but also to keep growing the food industry on the large. Herein, we discuss the insights of a comprehensive analysis of the chemical multiverse of food components (i.e., chemical space considering multiple representations) collected in a large public compound database with more than 70,000 compounds, including those completely characterized and quantified and expected by omics predictive elucidation. After comparing the food components with natural products and drugs approved for clinical use, we concluded that there is a large overlap between food components and natural products, which agrees with previous studies. Moreover, 426 food components in FooDB are also used for therapeutic treatment. Despite the fact that there is a considerable overlap between food components and natural products, food components are distinguished by the high proportion of acyclic compounds. Through an analysis of MCS, branching and unsaturation were observed for structurally similar clusters of food components. We also found that food components are structurally more complex than natural products and drugs approved for clinical use, as quantified by CSP3. By chirality, they have a median complexity between approved drugs and natural products. The fingerprint-based diversity of food components is lower than that of the reference databases, regardless of the nature of the fingerprint used (structural keys, radial fingerprints, or hybrid). However, the structural diversity of the commercial fraction of food components is very high. The chemical multiverse of food components is wider than that of natural products and approved drugs, covering common regions and spreading out of them. The most represented biosynthetic pathway in food components was fatty acids, which is in line with the structural and property characterization and guarantees follow-up studies addressing key features such as their enzymatic treatment, unsaturated enrichment, or interesterification using computational approaches.

This study is an example of repurposing chemoinformatic techniques typically used in drug discovery projects to analyze food components. It is expected that the field of food chemical informatics (also known as foodinformatics) will continue developing in the next several years.

## Data Availability

FooDB on its latest version was obtained from https://foodb.ca/downloads. The FDA set was downloaded from the DrugBank in the URL https://go.drugbank.com. The Universal Natural Product Database—Subset A (UNPD-A) was downloaded from the GitHub repository of the original publication, available in the URL https://github.com/DIFACQUIM/Natural-products-subsets-generation. Curated data sets as well as codes are freely available at https://github.com/DIFACQUIM/Food_chemicals_characterization.

## Supporting Information Available

Details of the comprehensive profiling of FooDB and reference compound collections analyzed in this work: descriptive statistics of properties and other chemoinformatic descriptors, predictions of the biosynthetic pathway, and visual representations of the chemical space (PDF)

## Abbreviations

CDF: cumulative distribution functions
COCONUT: COlleCtion of Open Natural ProdUcTs
CSP3: fraction of sp^3^ carbon atoms
ECFP: extended connectivity fingerprint
FDA: U.S. Food and Drug Administration
GRAS: Generally Recognized as Safe
HBA: hydrogen bond acceptors
HBD: hydrogen bond donors
IMP: invalid metabolic panaceas
IMPPAT: Indian Medicinal Plants, Phytochemistry And Therapeutics
logP: partition coefficient octanol/water
MACCS: Molecular ACCes System
MAP4: MinHashed atom-pair fingerprint up to a diameter of four bonds
MCS: maximum common substructure
MW: molecular weight
NP: natural products
NPL: natural product likeness
NSAID: nonsteroidal anti-inflammatory drugs
PAINS: pan-assay interference compounds
PCA: principal component analysis
RB: rotatable bonds
SMILES: Simplified Molecular Input Line Entry System
t-SNE: t-distributed stochastic neighbor embedding
UNPD: Universal Natural Product Database
UNPD-A: Universal Natural Product Database—Subset A
