# Supplementary material for: Chemical Multiverse and Diversity of Food Chemicals
Source: J Chem Inf Model. 2024 Feb 15;64(4):1229–44. doi: 10.1021/acs.jcim.3c01617 (PMC10900296; doi:10.1021/acs.jcim.3c01617)
Supplement: Supplementary file 1 — ci3c01617_si_001.pdf [file ci3c01617_si_001.pdf]

SUPPORTING INFORMATION

Chemical Multiverse and Diversity of Food Chemicals

Juan F. Avellaneda-Tamayo, Ana L. Chávez-Hernández, Diana L. Prado-Romero, José L. Medina-Franco*

DIFACQUIM Research Group, Department of Pharmacy, School of Chemistry, Universidad Nacional Autónoma de México, Avenida Universidad 3000, Mexico City 04510, Mexico

*Correspondence author: medinajl@unam.mx; Tel.: +52-55-5622-3899

Contents

		Page
**Table S1**	Descriptive statistics of physicochemical and constitutional descriptors computed for food components (FooDB), natural products (UNPD-A), FDA-approved drugs, and commercially available compounds from FooDB.	S3
**Table S2**	Descriptive statistics of complexity indexes for food components (FooDB), natural products (UNPD-A), FDA-approved drugs, and commercially available compounds from FooDB.	S9
**Figure S1**	Distribution of physicochemical properties and constitutional descriptors of interest among approved drugs, compounds of FooDB, commercially available compounds of FooDB, and natural products.	S10
**Figure S2**	Density plot of CSP3 vs. DataWarrior complexity index pairwise comparisons.	S12
**Figure S3**	Examples of chemical structures present in some clusters found in food components.	S13
**Figure S4**	Representative maximum substructures of natural products and FDA-approved drugs, computed for some clusters. n represents the number of molecules that share the substructure within the cluster.	S17
**Table S3**	Descriptive statistics of natural product-likeness scores computed for food components (FooDB), natural products (UNPD-A), FDA-approved drugs, and commercially available compounds from FooDB.	S18
**Table S4**	Descriptive statistics of similarity distribution computed for food components (FooDB), natural products (UNPD-A), FDA-approved drugs, and commercially available compounds from FooDB.	S19
**Table S5**	Summary of the food components profiling according to their biosynthetic pathway, superclass, and class (based on NPClassifier).	S20
**Table S6**	Summary of the natural products from UNPD-A profiling predicted according to their biosynthetic pathway, superclass, and class (based on NPClassifier).	S20

**Table S7**	Summary of the FDA-approved drugs profiling predicted according to their biosynthetic pathway, superclass, and class (based on NPClassifier).	S21
**Table S8**	Summary of the commercially available food components profiling, predicted according to their biosynthetic pathway, superclass, and class (based on NPClassifier).	S21
**Figure S5**	Chemical multiverse visualization of food components, and their comparison with natural products and approved drugs, using t-SNE and ECFP6 as molecular representations.	S22

Table S1. Descriptive statistics of physicochemical and constitutional descriptors computed for food components (FooDB), natural products (UNPD-A), FDA-approved drugs, and commercially available compounds from FooDB.

Descriptor	Data set	Number of compounds	mean	std[a]	min[b]	Q1[c]	median	Q3[d]	max[e]
Number of acidic atoms	FooDB	52856	0.086	0.545	0.000	0.000	0.000	0.000	32.000
	UNPD-A	14994	0.000	0.000	0.000	0.000	0.000	0.000	0.000
	FDA	2324	0.118	0.711	0.000	0.000	0.000	0.000	12.000
	FooDB purchasable	2422	0.000	0.000	0.000	0.000	0.000	0.000	0.000
Number of aromatic rings	FooDB	52856	0.315	0.887	0.000	0.000	0.000	0.000	20.000
	UNPD-A	14994	1.278	1.495	0.000	0.000	1.000	2.000	15.000
	FDA	2324	1.537	1.310	0.000	0.000	1.000	2.000	10.000
	FooDB purchasable	2422	0.976	1.040	0.000	0.000	1.000	1.000	8.000
Number of aromatic atoms	FooDB	52856	1.565	4.517	0.000	0.000	0.000	0.000	120.000
	UNPD-A	14994	6.190	7.428	0.000	0.000	6.000	12.000	84.000
	FDA	2324	7.634	6.735	0.000	0.000	6.000	12.000	60.000
	FooDB purchasable	2422	4.772	4.743	0.000	0.000	6.000	6.000	38.000
Number of basic atoms	FooDB	52856	0.041	0.211	0.000	0.000	0.000	0.000	5.000
	UNPD-A	14994	0.000	0.000	0.000	0.000	0.000	0.000	0.000
	FDA	2324	0.052	0.266	0.000	0.000	0.000	0.000	4.000
	FooDB purchasable	2422	0.011	0.111	0.000	0.000	0.000	0.000	2.000
Number of	FooDB	52856	0.001	0.073	0.000	0.000	0.000	0.000	7.000

bromine atoms	UNPD-A	14994	0.054	0.397	0.000	0.000	0.000	0.000	7.000
	FDA	2324	0.018	0.168	0.000	0.000	0.000	0.000	4.000
	FooDB purchasable	2422	0.017	0.288	0.000	0.000	0.000	0.000	7.000
Number of alicyclic rings of carbon	FooDB	52856	0.272	0.945	0.000	0.000	0.000	0.000	8.000
	UNPD-A	14994	0.960	1.484	0.000	0.000	0.000	2.000	10.000
	FDA	2324	0.511	1.132	0.000	0.000	0.000	0.000	6.000
	FooDB purchasable	2422	0.053	0.235	0.000	0.000	0.000	0.000	2.000
Number of aromatic rings of carbon	FooDB	52856	0.215	0.710	0.000	0.000	0.000	0.000	20.000
	UNPD-A	14994	0.963	1.273	0.000	0.000	0.000	2.000	14.000
	FDA	2324	1.032	1.000	0.000	0.000	1.000	2.000	10.000
	FooDB purchasable	2422	0.650	0.805	0.000	0.000	0.000	1.000	6.000
Number of chiral centers	FooDB	52856	2.731	4.937	0.000	1.000	1.000	2.000	75.000
	UNPD-A	14994	3.805	5.129	0.000	0.000	2.000	6.000	43.000
	FDA	2324	2.304	3.826	0.000	0.000	1.000	3.000	41.000
	FooDB purchasable	2422	0.000	0.000	0.000	0.000	0.000	0.000	0.000
Number of chlorine atoms	FooDB	52856	0.006	0.126	0.000	0.000	0.000	0.000	10.000
	UNPD-A	14994	0.039	0.343	0.000	0.000	0.000	0.000	10.000
	FDA	2324	0.203	0.592	0.000	0.000	0.000	0.000	6.000
	FooDB purchasable	2422	0.033	0.300	0.000	0.000	0.000	0.000	5.000

CSP3	FooDB	52856	0.756	0.225	0.000	0.667	0.820	0.939	1.000
	UNPD-A	14994	0.519	0.308	0.000	0.250	0.522	0.800	1.000
	FDA	2324	0.454	0.270	0.000	0.263	0.429	0.632	1.000
	FooDB purchasable	2422	0.445	0.339	0.000	0.143	0.364	0.800	1.000
Number of fluorine atoms	FooDB	52856	0.002	0.117	0.000	0.000	0.000	0.000	17.000
	UNPD-A	14994	0.001	0.054	0.000	0.000	0.000	0.000	3.000
	FDA	2324	0.280	0.961	0.000	0.000	0.000	0.000	14.000
	FooDB purchasable	2422	0.015	0.247	0.000	0.000	0.000	0.000	8.000
Fraction of rotatable bonds	FooDB	52856	0.648	0.292	0.000	0.438	0.804	0.857	0.952
	UNPD-A	14994	0.188	0.202	0.000	0.053	0.125	0.235	0.967
	FDA	2324	0.226	0.154	0.000	0.125	0.200	0.300	0.949
	FooDB purchasable	2422	0.306	0.264	0.000	0.100	0.222	0.500	0.941
Number of halogen atoms	FooDB	52856	0.010	0.198	0.000	0.000	0.000	0.000	17.000
	UNPD-A	14994	0.097	0.542	0.000	0.000	0.000	0.000	10.000
	FDA	2324	0.549	1.197	0.000	0.000	0.000	1.000	14.000
	FooDB purchasable	2422	0.069	0.516	0.000	0.000	0.000	0.000	8.000
HBA	FooDB	52856	6.754	4.918	0.000	5.000	6.000	6.000	104.000
	UNPD-A	14994	5.576	4.953	0.000	2.000	4.000	7.000	53.000
	FDA	2324	5.287	4.612	0.000	3.000	4.000	6.000	59.000
	FooDB	2422	2.524	1.705	0.000	1.000	2.000	3.000	10.000

	purchasable								
HBD	FooDB	52856	1.593	3.519	0.000	0.000	0.000	2.000	73.000
	UNPD-A	14994	2.506	3.173	0.000	0.000	2.000	3.000	36.000
	FDA	2324	2.444	3.664	0.000	1.000	2.000	3.000	56.000
	FooDB purchasable	2422	0.957	1.159	0.000	0.000	1.000	2.000	6.000
Number of heavy atoms	FooDB	52856	51.997	22.982	0.000	35.000	58.000	67.000	322.000
	UNPD-A	14994	26.377	13.903	1.000	18.000	24.000	32.000	135.000
	FDA	2324	26.680	18.861	1.000	17.000	23.000	31.000	291.000
	FooDB purchasable	2422	13.939	7.578	4.000	9.000	12.000	17.000	69.000
Number of heteroatoms	FooDB	52856	7.388	5.817	0.000	6.000	6.000	6.000	126.000
	UNPD-A	14994	6.020	5.081	0.000	3.000	5.000	7.000	53.000
	FDA	2324	7.502	6.967	0.000	4.000	6.000	9.000	106.000
	FooDB purchasable	2422	3.018	2.057	0.000	2.000	2.000	4.000	15.000
Number of alicyclic rings with heteroatoms	FooDB	52856	0.314	0.983	0.000	0.000	0.000	0.000	30.000
	UNPD-A	14994	0.857	1.259	0.000	0.000	0.000	1.000	21.000
	FDA	2324	0.731	1.211	0.000	0.000	0.000	1.000	30.000
	FooDB purchasable	2422	0.066	0.281	0.000	0.000	0.000	0.000	3.000
Number of aromatic rings with heteroatomx	FooDB	52856	0.100	0.378	0.000	0.000	0.000	0.000	8.000
	UNPD-A	14994	0.315	0.604	0.000	0.000	0.000	1.000	10.000
	FDA	2324	0.505	0.773	0.000	0.000	0.000	1.000	6.000

	FooDB purchasable	2422	0.326	0.533	0.000	0.000	0.000	1.000	3.000
Number of iodine atoms	FooDB	52856	0.001	0.051	0.000	0.000	0.000	0.000	4.000
	UNPD-A	14994	0.003	0.069	0.000	0.000	0.000	0.000	3.000
	FDA	2324	0.048	0.402	0.000	0.000	0.000	0.000	6.000
	FooDB purchasable	2422	0.004	0.118	0.000	0.000	0.000	0.000	4.000
ClogP	FooDB	52856	12.222	8.059	-30.874	3.977	14.868	18.727	33.828
	UNPD-A	14994	2.940	3.017	-18.528	1.464	2.868	4.325	24.432
	FDA	2324	2.271	2.871	-24.035	0.697	2.547	3.940	18.551
	FooDB purchasable	2422	2.422	2.385	-6.970	1.170	2.031	3.094	21.110
MW	FooDB	52856	736.410	326.871	1.008	502.557	821.366	933.217	4628.234
	UNPD-A	14994	371.937	196.432	16.043	246.306	330.294	445.595	1887.281
	FDA	2324	387.381	271.961	12.011	247.952	337.365	447.143	4113.641
	FooDB purchasable	2422	198.177	109.008	54.048	132.115	168.137	230.306	975.663
Number of nitrogen atoms	FooDB	52856	0.313	1.126	0.000	0.000	0.000	0.000	61.000
	UNPD-A	14994	0.485	1.214	0.000	0.000	0.000	0.000	18.000
	FDA	2324	2.542	3.274	0.000	1.000	2.000	3.000	51.000
	FooDB purchasable	2422	0.458	0.853	0.000	0.000	0.000	1.000	6.000
Number of oxygen atoms	FooDB	52856	6.845	5.203	0.000	5.000	6.000	6.000	104.000
	UNPD-A	14994	5.376	5.053	0.000	2.000	4.000	7.000	53.000

	FDA	2324	4.040	4.454	0.000	2.000	3.000	5.000	59.000
	FooDB purchasable	2422	2.354	1.901	0.000	1.000	2.000	3.000	10.000
Number of ring systems	FooDB	52856	0.902	1.897	0.000	0.000	0.000	1.000	32.000
	UNPD-A	14994	3.095	2.192	0.000	2.000	3.000	4.000	21.000
	FDA	2324	2.779	1.977	0.000	1.000	3.000	4.000	30.000
	FooDB purchasable	2422	1.095	1.105	0.000	0.000	1.000	2.000	8.000
Number of rotable bonds	FooDB	52856	35.252	21.653	0.000	9.000	43.000	51.000	148.000
	UNPD-A	14994	4.742	6.019	0.000	1.000	3.000	6.000	59.000
	FDA	2324	5.984	7.633	0.000	2.000	4.000	7.000	149.000
	FooDB purchasable	2422	4.240	6.202	0.000	1.000	2.000	5.000	59.000
TPSA	FooDB	52856	103.531	90.325	0.000	78.900	78.900	93.060	2093.550
	UNPD-A	14994	90.782	82.741	0.000	40.460	69.670	112.050	877.360
	FDA	2324	95.725	106.375	0.000	43.370	74.600	110.768	1693.140
	FooDB purchasable	2422	43.826	32.083	0.000	20.230	37.300	61.810	208.060

[a] std: standard deviation.
[b] min: minimum value.
[c] Q1: value under which 25% of data points are found in increasing order.
[d] Q3: value under which 75% of data points are found in increasing order.
[e] max: maximum value.

Table S2. Descriptive statistics of complexity indexes for food components (FooDB), natural products (UNPD-A), FDA-approved drugs, and commercially available compounds from FooDB.

Descriptor	Data set	Number of compounds	mean	std[a]	min[b]	Q1[c]	median	Q3[d]	max[e]
CSP3	FooDB	68648.0	0.792	0.215	0.000	0.712	0.892	0.943	1.000
	UNPD-A	14987.0	0.519	0.308	0.000	0.250	0.524	0.800	1.000
	FDA	2320.0	0.454	0.270	0.000	0.263	0.429	0.632	1.000
	FooDB purchasable	3329.0	0.501	0.332	0.000	0.200	0.462	0.833	1.000
Datawarrior complexity	FooDB	68648.0	0.697	0.126	0.000	0.654	0.666	0.687	1.318
	UNPD-A	14987.0	0.832	0.191	0.000	0.728	0.873	0.969	1.281
	FDA	2320.0	0.780	0.197	0.000	0.681	0.809	0.912	1.181
	FooDB purchasable	3329.0	0.629	0.177	0.000	0.515	0.641	0.738	1.117

[a] std: standard deviation.
[b] min: minimum value.
[c] Q1: value under which 25% of data points are found in increasing order.
[d] Q3: value under which 75% of data points are found in increasing order.
[e] max: maximum value.

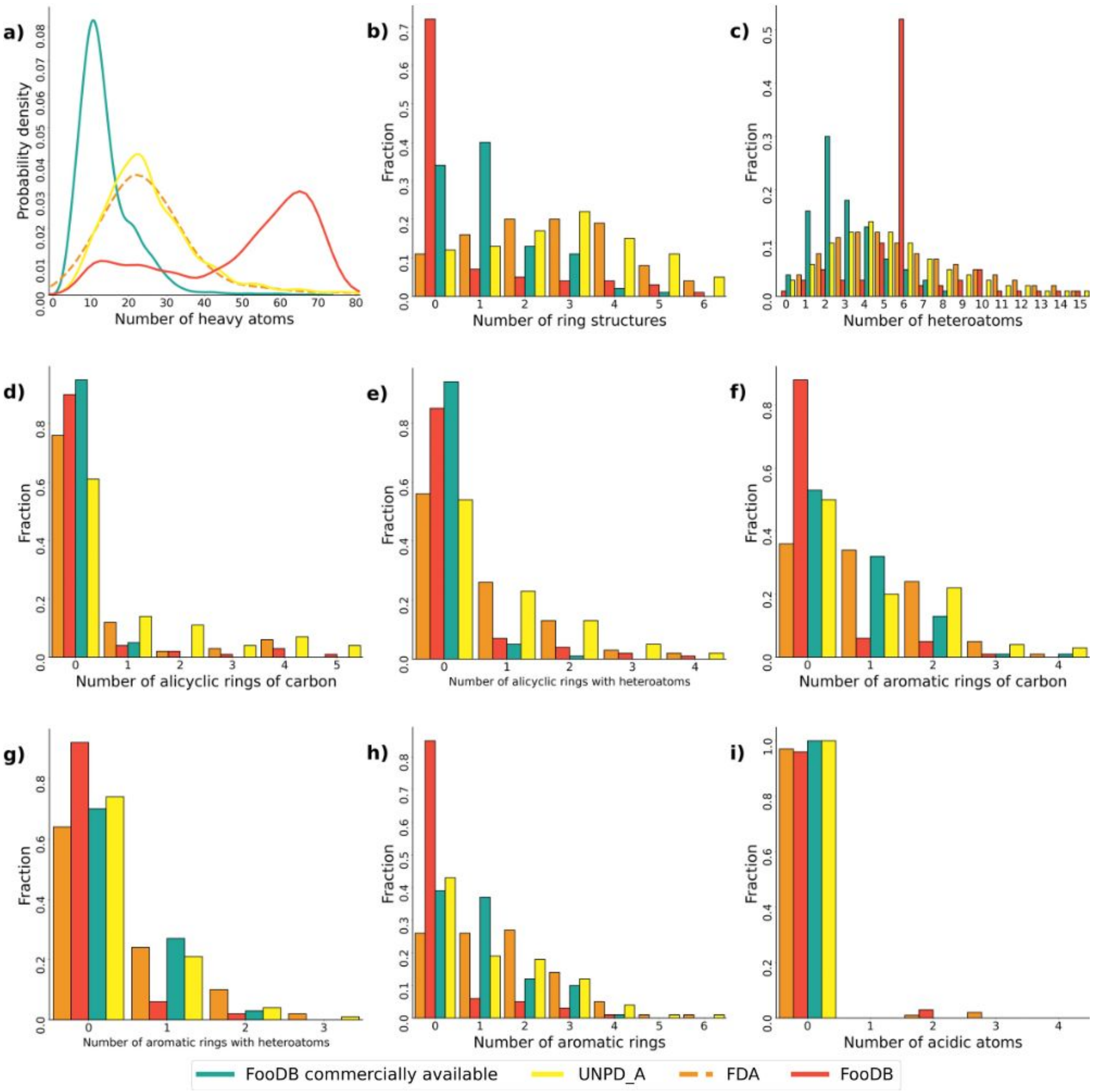

Figure S1. Distribution of physicochemical properties and constitutional descriptors of interest among approved drugs (orange), compounds of FooDB (red), commercially available compounds of FooDB (green), and natural products (UNPD-A; yellow): a) number of heavy atoms, b) number of ring structures, c) number of heteroatoms, d) number of alicyclic rings of carbon, e) number of alicyclic rings with heteroatoms, f) number of aromatic rings of carbon, g) number of aromatic rings with heteroatoms, h) number of aromatic rings, i) number of acidic atoms. Dotted lines are used for ease of visualization.

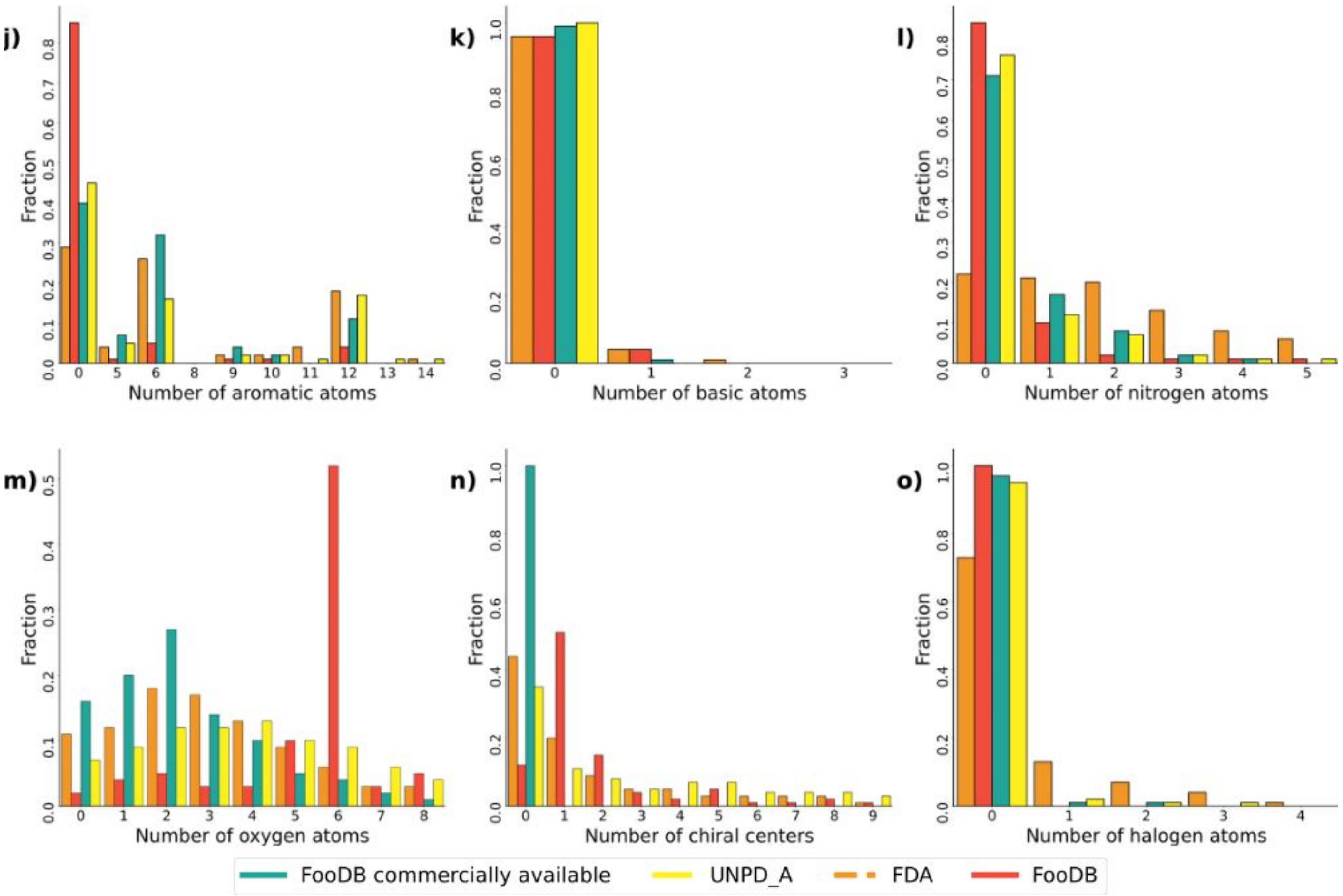

Figure S1 (continued). Distribution of physicochemical properties and constitutional descriptors of interest among approved drugs (orange), compounds of FooDB (red), commercially available compounds of FooDB (green), and natural products (UNPD-A; yellow): j) number of aromatic atoms, k) number of basic atoms, l) number of nitrogen atoms, m), number of oxygen atoms, n) number of chiral centers, o) number of halogen atoms (cont.).

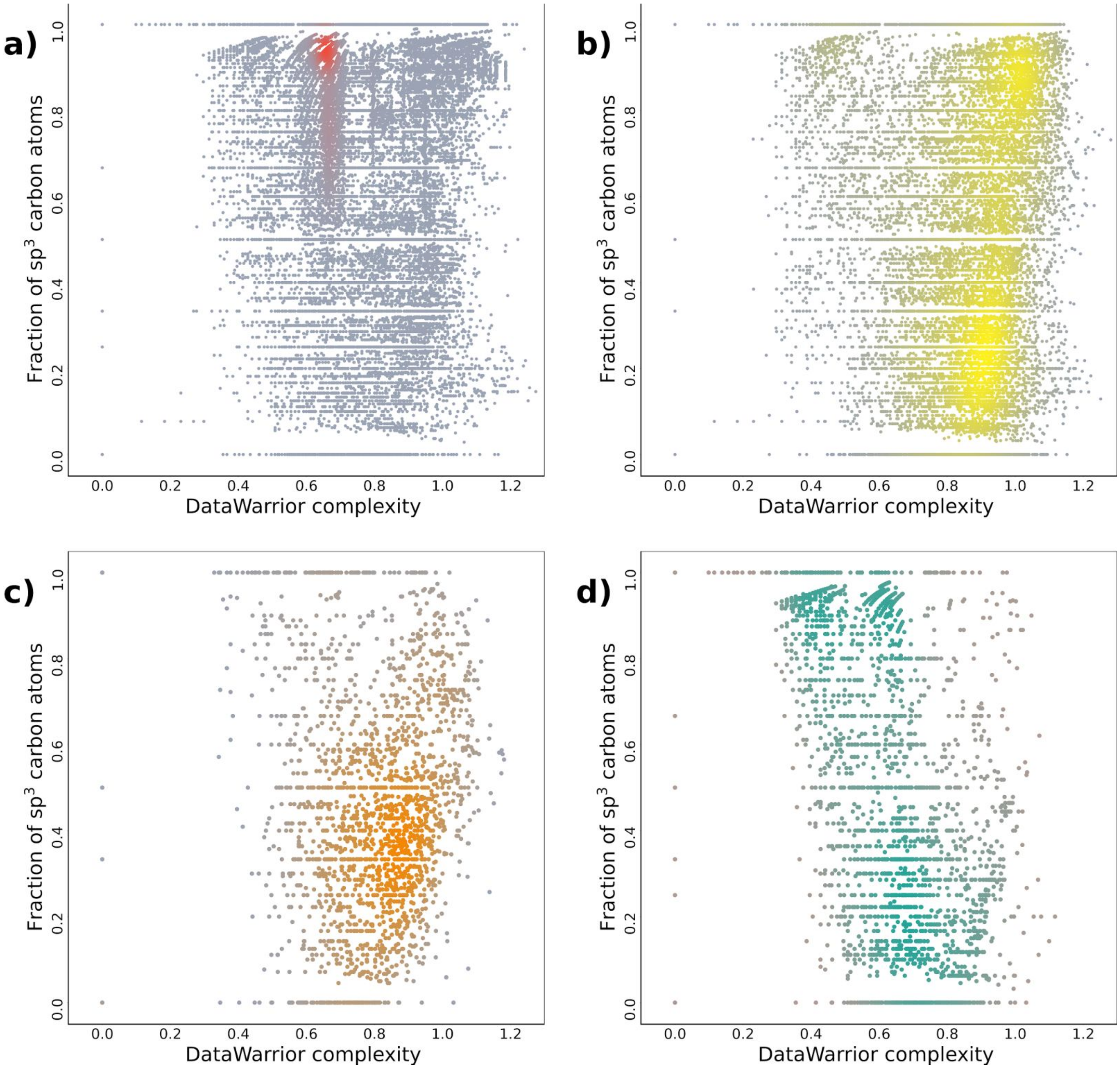

Figure S2. Density plot of CSP3 vs. DataWarrior complexity index pairwise comparison, computed for a) food components (FooDB, gray-red), b) natural products (UNPD-A, gray-yellow), b) FDA-approved drugs (gray-orange), and d) commercially available compounds from FooDB (gray-green). The density of data points is represented in a continuous scale from denser (colored), to less dense (gray).

Cluster 1

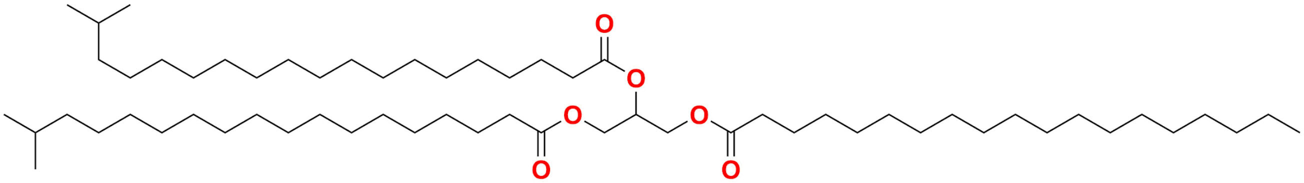

FDB082633

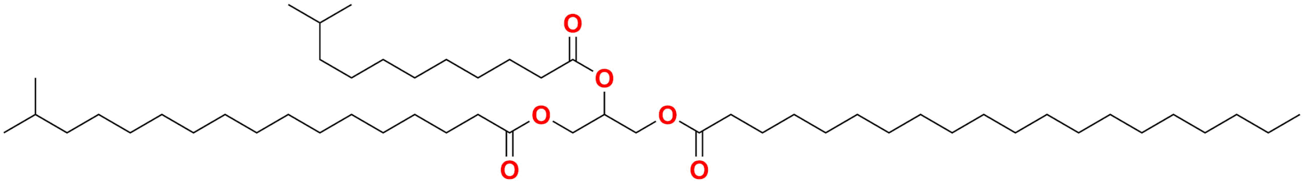

FDB034855

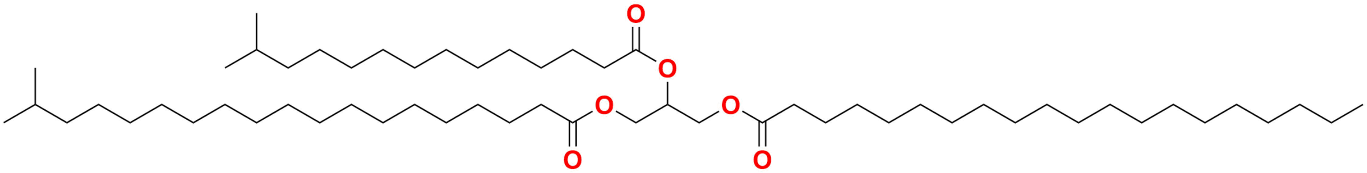

FDB034856

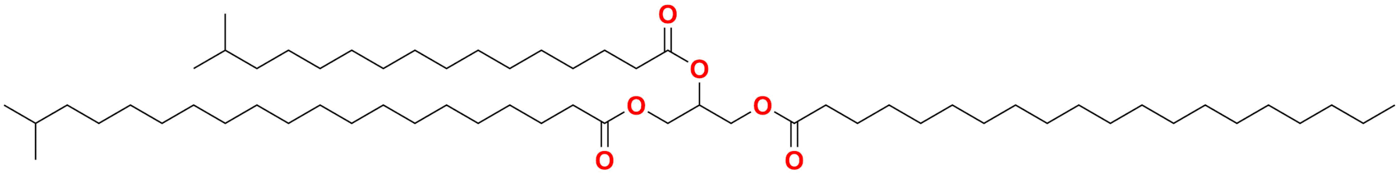

FDB034865

Cluster 2

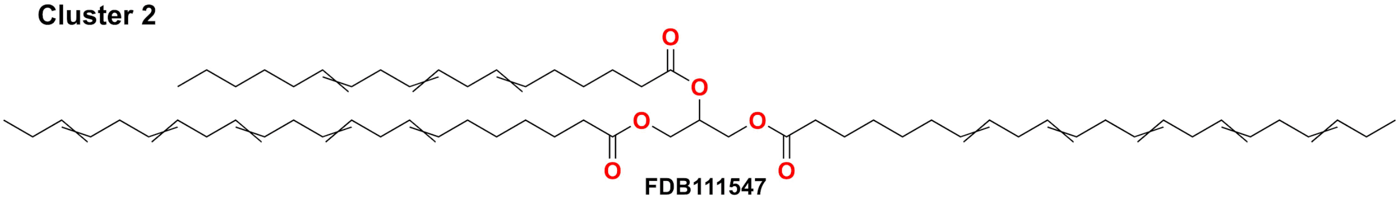

FDB111547

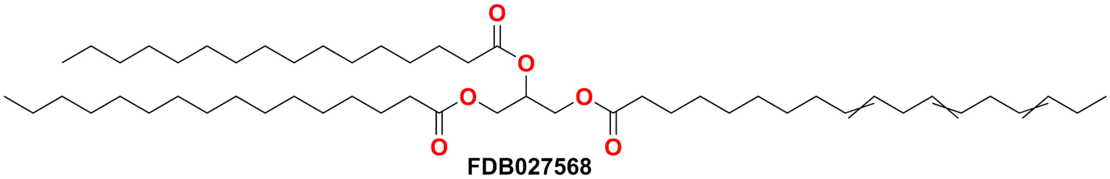

FDB027568

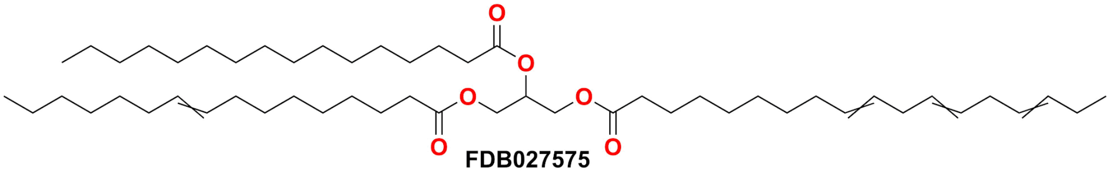

FDB027575

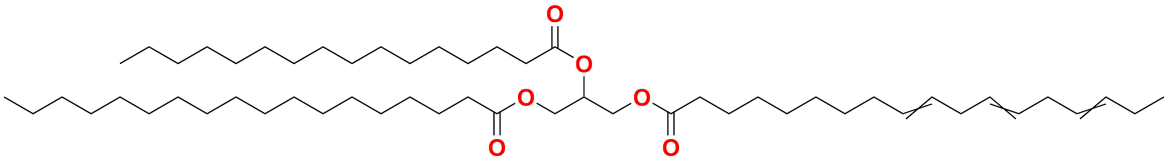

FDB027583

Figure S3. Examples of chemical structures present in some clusters that are found in food components.

Cluster 3

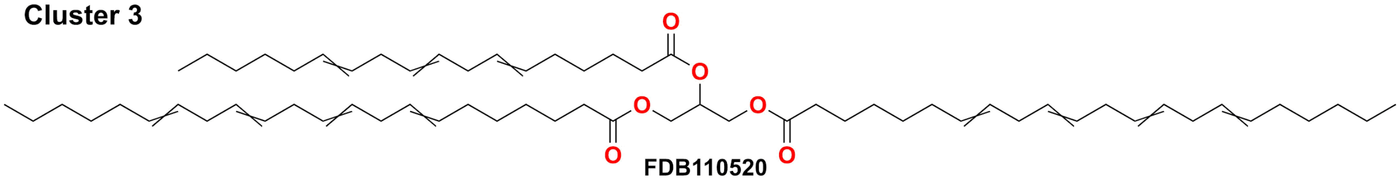

FDB110520

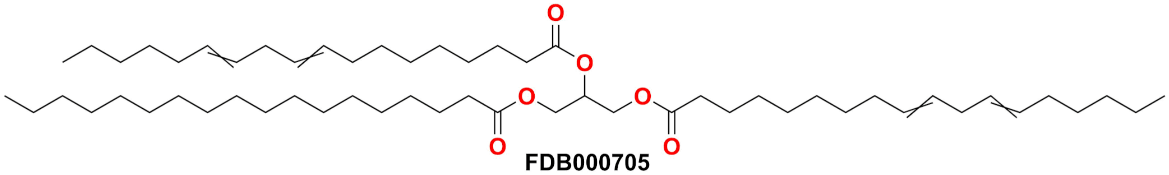

FDB000705

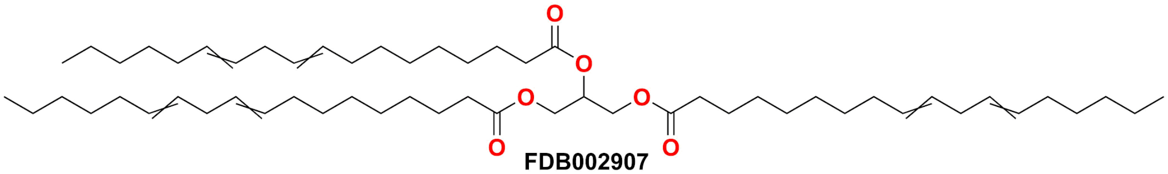

FDB002907

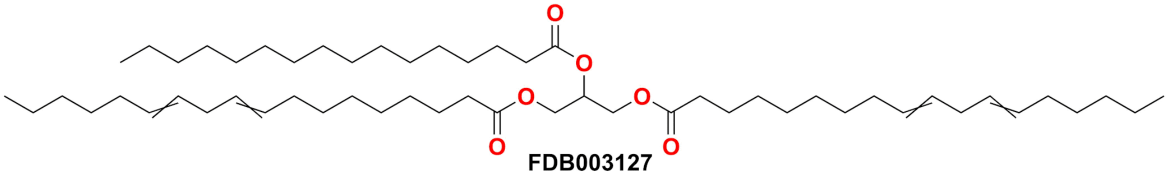

FDB003127

Cluster 4

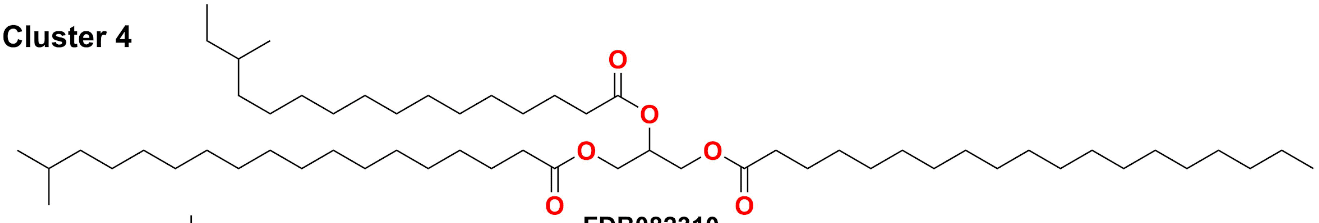

FDB082310

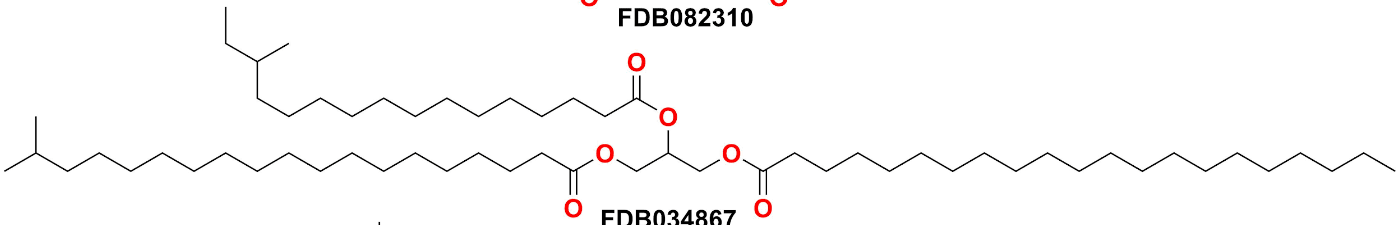

FDB034867

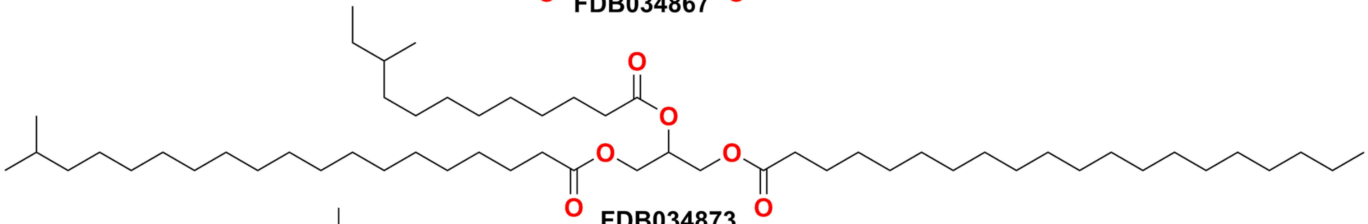

FDB034873

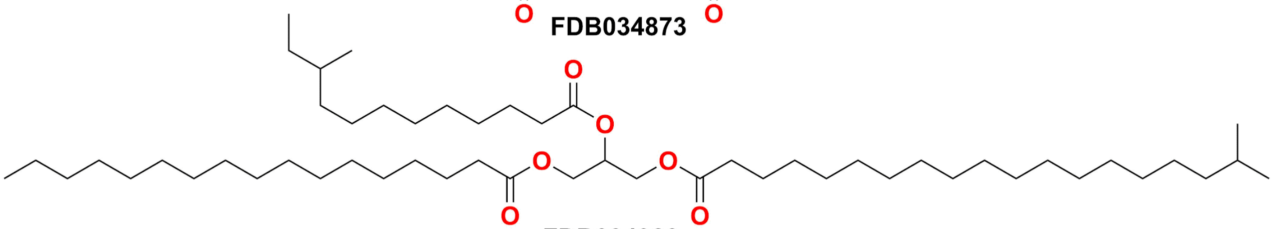

FDB034923

Figure S3. Examples of chemical structures present in some clusters that are found in food components (continued).

Cluster 50

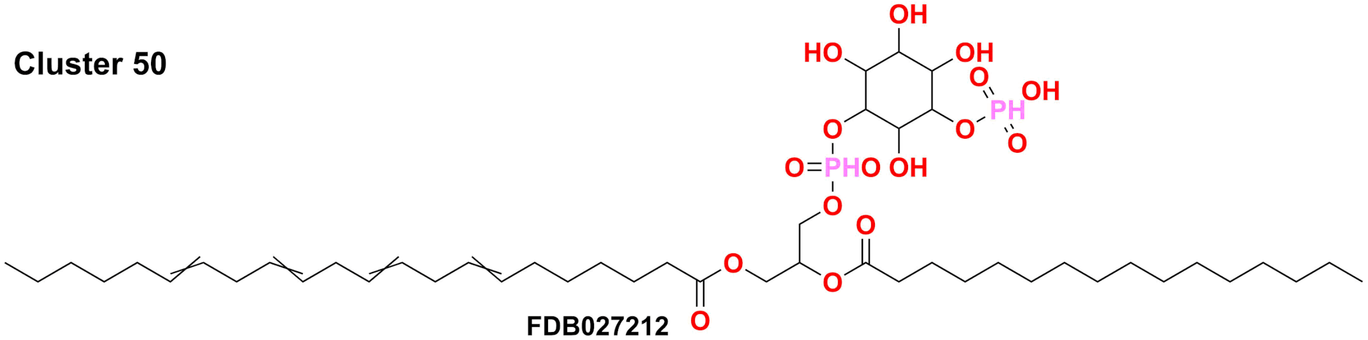

FDB027212

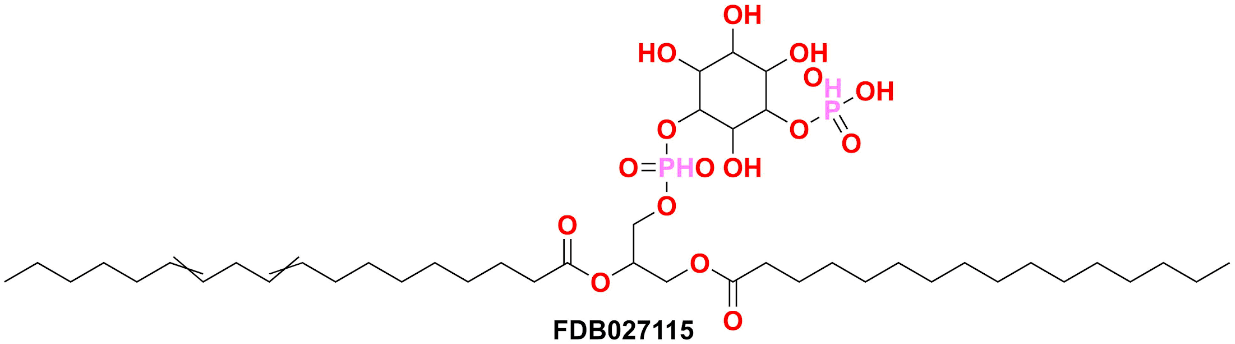

FDB027115

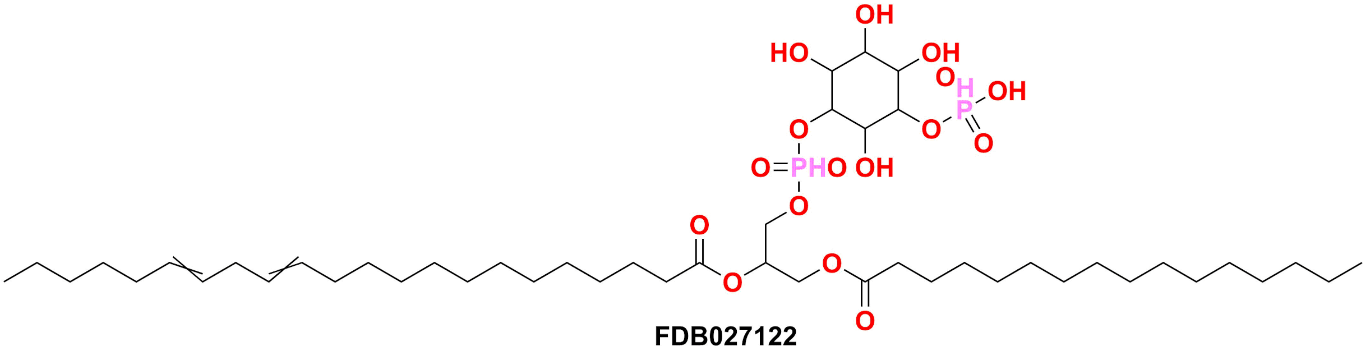

FDB027122

Figure S3. Examples of chemical structures present in some clusters that are found in food components (continued).

Cluster 100

FDB028070

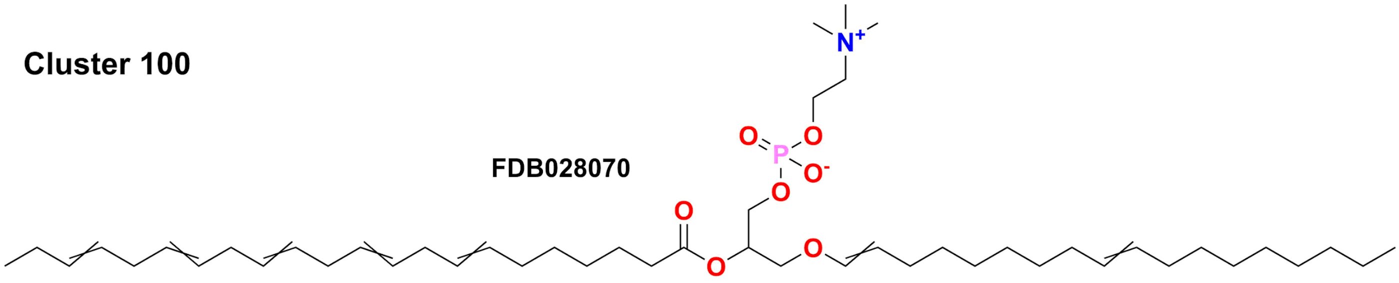

FDB027969

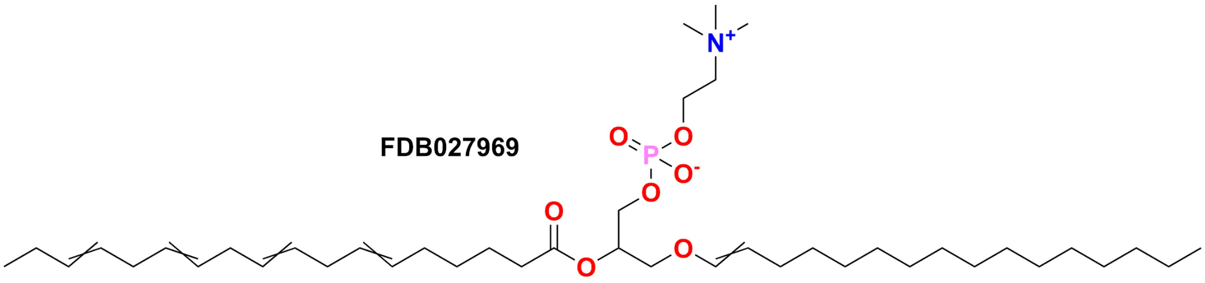

FDB027983

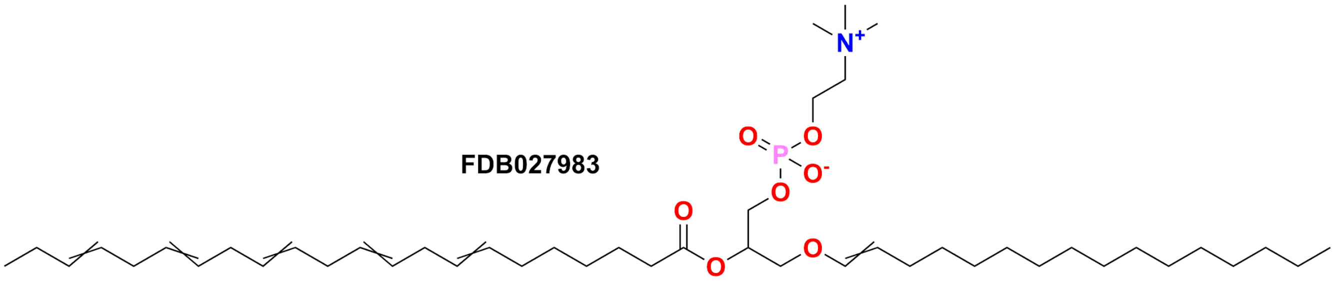

Figure S3. Examples of chemical structures present in some clusters that are found in food components (continued).

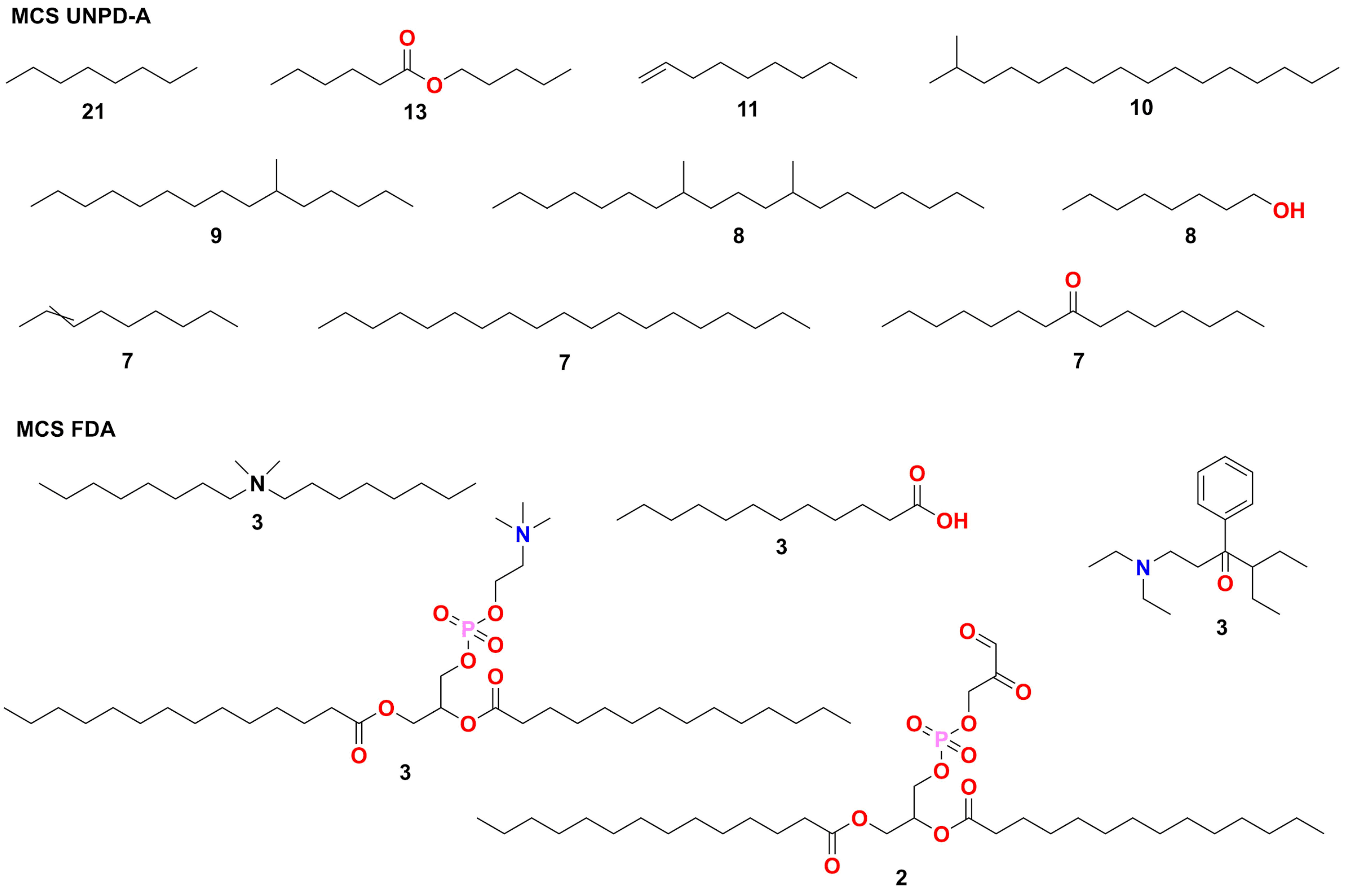

Figure S4. Representative maximum substructures of natural products (UNPD-A) and FDA-approved drugs, computed for some clusters. The number below each structure is the number of molecules that share the substructure within each cluster.

Table S3. Descriptive statistics of natural product-likeness scores computed for food components (FooDB), natural products (UNPD-A), FDA-approved drugs, and commercially available compounds from FooDB.

Data set	FooDB	UNPD-A	FDA	FooDB purchasable
count	68,658	14,994	2,324	3,330
mean	0.67	1.51	0.02	0.51
std[a]	0.71	1.05	1.08	1.02
min[b]	-2.96	-2.15	-2.50	-2.96
Q1[c]	0.32	0.75	-0.79	-0.08
median	0.44	1.51	-0.10	0.44
Q3[d]	0.64	2.32	0.63	1.09
max[e]	3.94	4.08	3.93	3.93

[a] std: standard deviation.
[b] min: minimum value.
[c] Q1: value under which 25% of data points are found in increasing order.
[d] Q3: value under which 75% of data points are found in increasing order.
[e] max: maximum value.

Table S4. Descriptive statistics of similarity distribution computed for food components (FooDB), natural products (UNPD-A), FDA-approved drugs, and commercially available compounds from FooDB.[d]

Fingerprint	Data set	number of pairwise comparisons	mean	std[a]	Q1[b]	median	Q3[c]
MACCS keys 166 bits	FooDB	12497500	0.645	0.306	0.354	0.619	0.933
	UNPD-A	112402521	0.350	0.172	0.222	0.341	0.464
	FDA	2699326	0.300	0.144	0.200	0.295	0.395
	FooDB purchasable	5542785	0.256	0.172	0.132	0.222	0.343
ECFP4 1024 bits	FooDB	12497500	0.437	0.320	0.100	0.474	0.692
	UNPD-A	112402521	0.098	0.057	0.060	0.091	0.127
	FDA	2699326	0.096	0.051	0.065	0.094	0.124
	FooDB purchasable	5542785	0.115	0.101	0.054	0.091	0.143
ECFP6 1024 bits	FooDB	12497500	0.403	0.313	0.084	0.418	0.640
	UNPD-A	112402521	0.081	0.043	0.053	0.077	0.104
	FDA	2699326	0.081	0.042	0.056	0.080	0.104
	FooDB purchasable	5542785	0.094	0.085	0.047	0.075	0.115
MAP4 1024 bits	FooDB	12497500	0.225	0.219	0.009	0.199	0.395
	UNPD-A	112402521	0.010	0.020	0.000	0.002	0.012
	FDA	2699326	0.008	0.019	0.000	0.003	0.010
	FooDB purchasable	5542785	0.019	0.051	0.000	0.001	0.016

[a] std: standard deviation.
[b] Q1: value under which 25% of data points are found in increasing order.
[c] Q3: value under which 75% of data points are found in increasing order.
[d] Minimum and maximum values were 0, and 1 respectively for all data sets and all different representations.

Table S5. Summary of the food components profiling according to their biosynthetic pathway, superclass, and class (based on NPClassifier).

**Pathway**	**Count**		**Percentage**
Fatty acids	51821		78,9
Terpenoids	5255		8,0
Shikimates and Phenylpropanoids	3867		5,9
Alkaloids	1695		2,6
Amino acids and Peptides	1396		2,1
Polyketides	857		1,3
Carbohydrates	750		1,1
**SuperClass**	**Percentage**	**Class**	**Percentage**
Glycerolipids	75,4	Triacylglycerols	70,2
Flavonoids	2,2	Diacylglycerols	3,0
Steroids	1,9	Dipeptides	1,0
Triterpenoids	1,9	Wax monoesters	0,8
Small peptides	1,7	Flavonols	0,8
Fatty acyls	1,5	Oleanane triterpenoids	0,8
Sesquiterpenoids	1,5	Aminoacids	0,7
Monoterpenoids	1,2	Flavones	0,6
Fatty esters	1,1	Fatty alcohols	0,6
Others	11,8	Others	21,6

Table S6. Summary of the natural products from UNPD-A profiling predicted according to their biosynthetic pathway, superclass, and class (based on NPClassifier).

**Pathway**	**Counts**		**Percentage**
Terpenoids	4774		32.8
Shikimates and Phenylpropanoids	3599		24.7
Alkaloids	2407		16.5
Polyketides	1619		11.1
Fatty acids	1407		9.7
Amino acids and Peptides	471		3.2
Carbohydrates	288		2.0
**SuperClass**	**Percentage**	**Class**	**Percentage**
Sesquiterpenoids	10.7	Flavones	2.7
Flavonoids	8.6	Hydrocarbons	3.2
Diterpenoids	7.8	Flavonols	1.9
Triterpenoids	6.7	Fatty alcohols	1.9
Fatty acyls	5.6	Oleanane triterpenoids	1.6
Tryptophan alkaloids	4.8	Aminoacids	1.4
Steroids	4.4	Germacrane sesquiterpenoids	1.3
Tyrosine alkaloids	3.2	Guaiane sesquiterpenoids	1.3
Monoterpenoids	3.2	Eudesmane sesquiterpenoids	1.3
Others	45.2	Others	83.5

Table S7. Summary of the FDA-approved drugs profiling predicted according to their biosynthetic pathway, superclass, and class (based on NPClassifier).

Pathway	Counts		Percentage
Alkaloids	991		51.2
Amino acids and Peptides	283		14.6
Terpenoids	207		10.7
Shikimates and Phenylpropanoids	201		10.4
Fatty acids	92		4.8
Polyketides	84		4.3
Carbohydrates	76		3.9
**SuperClass**	**Percentage**	**Class**	**Percentage**
Steroids	10.9	Steroids	5.5
Tryptophan alkaloids	10.3	Tryptophan alkaloids	5.1
Small peptides	10.2	Small peptides	5.1
Pseudoalkaloids	7.7	Pseudoalkaloids	3.9
Tyrosine alkaloids	7.2	Tyrosine alkaloids	3.6
Nicotinic acid alkaloids	4.9	Aminoacids	3.6
Oligopeptides	4.8	Pregnane steroids	2.8
β-lactams	4.5	Nicotinic acid alkaloids	2.4
Anthranilic acid alkaloids	3.9	Oligopeptides	2.4
Others	35.7	Others	65.5

Table S8. Summary of the commercially available food components profiling, predicted according to their biosynthetic pathway, superclass, and class (based on NPClassifier).

Pathway	Counts		Percentage
Fatty acids	1159		36.9
Shikimates and Phenylpropanoids	855		27.2
Alkaloids	507		16.1
Terpenoids	289		9.2
Amino acids and Peptides	161		5.1
Polyketides	137		4.4
Carbohydrates	35		1.1
**SuperClass**	**Percentage**	**Class**	**Percentage**
Fatty esters	11.8	Fatty esters	5.8
Fatty acyls	10.7	Wax monoesters	5.6
Glycerolipids	8.8	Fatty acyls	5.3
Fatty Acids and Conjugates	7.8	Glycerolipids	4.3
Flavonoids	7.3	Fatty Acids and Conjugates	3.8
Monoterpenoids	5.6	Flavonoids	3.6
Phenolic acids (C6-C1)	4.8	Triacylglycerols	3.5
Phenylpropanoids (C6-C3)	4.2	Monoterpenoids	2.8
Small peptides	3.5	Phenolic acids (C6-C1)	2.4
Others	35.3	Others	62.9

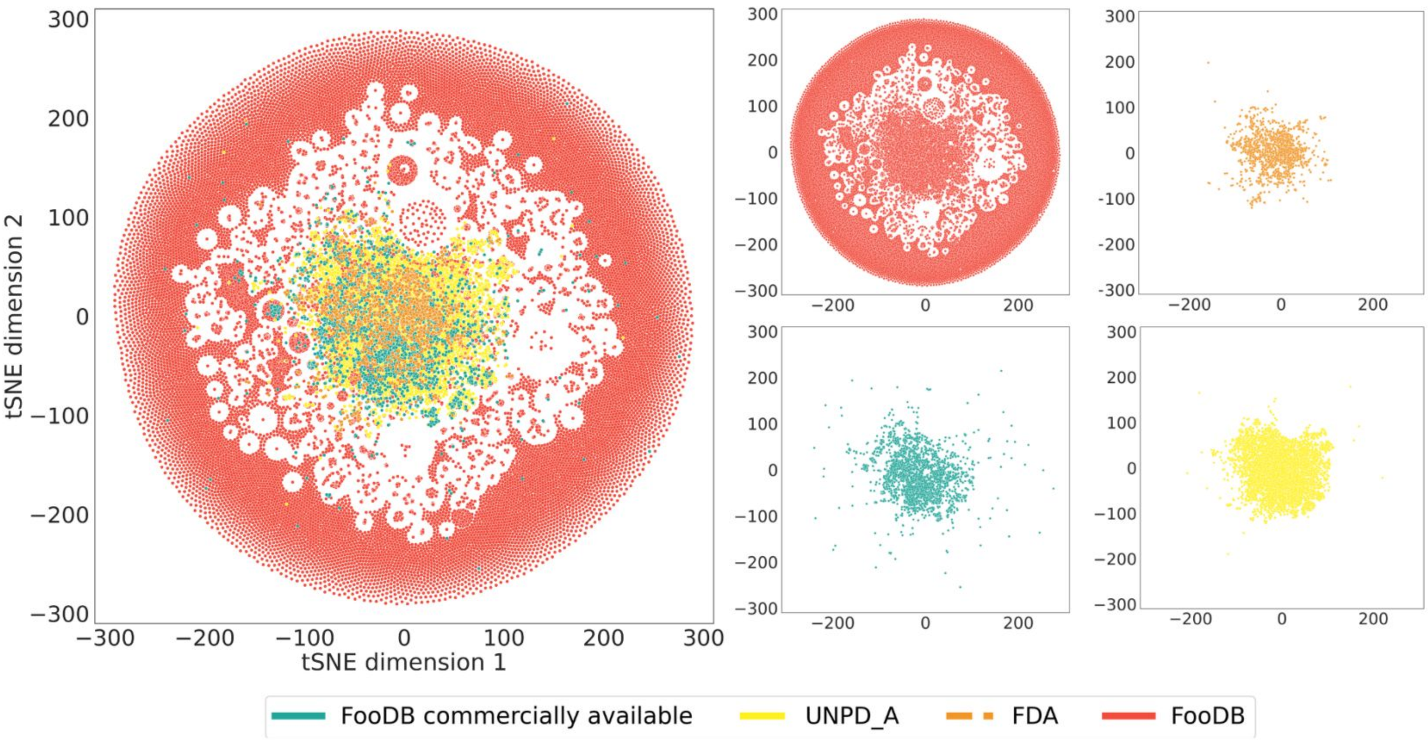

Figure S5. Chemical multiverse visualization of food components, and their comparison with natural products and approved drugs, using t-SNE and ECFP6 as molecular representations.
